# Simultaneous Enrichment of Cysteine-containing Peptides and Phosphopeptides Using a Cysteine-specific Phosphonate Adaptable Tag (CysPAT) in Combination with titanium dioxide (TiO_2_) Chromatography*[Fn FN2]

**DOI:** 10.1074/mcp.M115.054551

**Published:** 2016-10

**Authors:** Honggang Huang, Martin Haar Petersen, Maria Ibañez-Vea, Pernille S. Lassen, Martin R. Larsen, Giuseppe Palmisano

**Affiliations:** From the ‡Department of Biochemistry and Molecular Biology, University of Southern Denmark;; §The Danish Diabetes Academy, Odense, Denmark;; ¶Institute of Molecular Medicine, Cancer & Inflammation Research, University of Southern Denmark;; ‖Department of Parasitology, ICB, University of São Paulo, Brazil

## Abstract

Cysteine is a rare and conserved amino acid involved in most cellular functions. The thiol group of cysteine can be subjected to diverse oxidative modifications that regulate many physio-pathological states. In the present work, a Cysteine-specific Phosphonate Adaptable Tag (CysPAT) was synthesized to selectively label cysteine-containing peptides (Cys peptides) followed by their enrichment with titanium dioxide (TiO_2_) and subsequent mass spectrometric analysis. The CysPAT strategy was developed using a synthetic peptide, a standard protein and subsequently the strategy was applied to protein lysates from Hela cells, achieving high specificity and enrichment efficiency. In particular, for Cys proteome analysis, the method led to the identification of 7509 unique Cys peptides from 500 μg of HeLa cell lysate starting material. Furthermore, the method was developed to simultaneously enrich Cys peptides and phosphorylated peptides. This strategy was applied to SILAC labeled Hela cells subjected to 5 min epidermal growth factor (EGF) stimulation. In total, 10440 unique reversibly modified Cys peptides (3855 proteins) and 7339 unique phosphopeptides (2234 proteins) were simultaneously identified from 250 μg starting material. Significant regulation was observed in both phosphorylation and reversible Cys modification of proteins involved in EGFR signaling. Our data indicates that EGF stimulation can activate the well-known phosphorylation of EGFR and downstream signaling molecules, such as mitogen-activated protein kinases (MAPK1 and MAPK3), however, it also leads to substantial modulation of reversible cysteine modifications in numerous proteins. Several protein tyrosine phosphatases (PTPs) showed a reduction of the catalytic Cys site in the conserved putative phosphatase HC(X)5R motif indicating an activation and subsequent de-phosphorylation of proteins involved in the EGF signaling pathway. Overall, the CysPAT strategy is a straight forward, easy and promising method for studying redox proteomics and the simultaneous enrichment strategy offers an excellent solution for characterization of cross-talk between phosphorylation and redox induced reversible cysteine modifications.

Cysteine (Cys) is the second rarest amino acid, after tryptophan, with 214,000 Cys encoded by the human genome (1). However, it is highly conserved and widely present in more than 97% of human proteins suggesting its important regulatory role in biological systems (2). The thiol group (R-SH) of Cys residues can function as reversible redox switches in proteins through oxidative post-translational modifications (oxPTMs)^1^ by reactive species in the cell. The common oxPTMs of Cys include reversible disulfides, thiosulfinates, S-glutathionylation, sulfenic acids, sulfenamides, sulfinamides, S-nitrosylation, and irreversible oxidative forms such as sulfinic acids, sulfonic acids, and sulfonamides (3). The different reversible oxPTMs of Cys can function as a binary switch to regulate protein structure, activity, interactions and localization, and reflects the cellular redox state. The improper balance of the cellular redox state is related to various diseases such as diabetes, cardiovascular disease, neurodegenerative disorders and cancer (4).

The challenges associated with the characterization of the reversible Cys modifications, which are often substoichiometric and labile has prompted toward the development of many analytical strategies (2, 3). Two-dimensional gel-based fluorescent detection of reversible oxPTMs of Cys residues have complemented large scale mass spectrometry-based analyses (5). Site-directed characterization of Cys residues has been largely based on reaction of the Cys residues with reagents bearing an enrichment moiety by alkylation or reduction switch. The first large scale study of Cys peptides was achieved using isotope coded affinity tags (ICAT) with a biotin tag (6). However, the strong affinity constant between biotin/avidin limits the fully elution of labeled peptides from the resin. Moreover, the large biotin tag generates fragments in the tandem mass spectrometric (MS/MS) analysis, complicating the interpretation of spectra and subsequent peptide identification (7). Nowadays photo and chemical-cleavable ICAT alternatives have been reported (8, 9). The biotin affinity tag was also used in the biotin switch assay to study Cys S-nitrosylation modifications (10). The chemical workflow adopted in the biotin switch assay served as guide to develop several enrichment methods toward oxidative modified Cys peptides (11). It should be noted that the biotin switch assay is an indirect method and recently novel chemicals have been explored to directly tag oxidatively modified cysteines (12, 13). Cys reactive tandem mass tag (Cys-TMT) switch assay has been developed to identify and quantify S-nitrosylation in biological sample (14, 15). The enrichment in the Cys-TMT method is based on an anti-TMT antibody resin. The concept of “fluorous proteomics” is based on peptide tagging with perfluorinated moieties and subsequent enrichment by solid-phase extraction using a fluorous-functionalized stationary phase (16). The versatility of fluorous tags was shown toward different PTMs such as primary amine, phosphopeptides and cysteine-containing peptides (16). However, recovery of enriched labeled peptides from the fluorous solid-phase media is in the range of 50–55% (16). Recently, thiol-affinity enrichment of Cys peptides was introduced (17) and it employs thiol-reactive resins to capture and enrich cysteines with high specificity and efficiency (17–19).

Titanium dioxide (TiO_2_) chromatographic resin is widely used for enrichment of phosphorylated peptides with high efficiency and specificity (20, 21). the enrichment mechanism is the adsorption of phospho-group to the surface of TiO_2_ by forming a bridging bidentate surface complex (22). Recently, we introduced the concept of using phospho-based adaptable tags (PATs) in combination with TiO_2_ enrichment for identification and characterization of specific PTMs and applied the concept for the enrichment of O-GlcNAcylated peptides using a click chemistry-based phosphate tag (23, 24). Later, N-terminal peptide enrichment using phosphor-tagging and TiO_2_-based enrichment was shown (25). The PAT tags contain a specific reactive group and a phospho-group (*e.g.* phosphate or phosphonate), the reactive group can chemo-selectively react with the target chemical group in proteins or peptides, and the phospho-group enables the enrichment using TiO_2_ or other phospho-enrichment resins.

In this study we developed a novel Cysteine-specific Phosphonate Adaptable Tag (CysPAT) for enrichment and characterization of Cys peptides and reversible modified Cys peptides in combination with differential reduction and alkylation. The CysPAT was synthesized by a reaction between N-Succinimidyl iodoacetate (SIA) and 2-aminoethylphosphonic acid (2-AEP). The synthesized tag was used to selectively label Cys peptides with a phosphonate tag before enrichment with TiO_2_ and subsequent mass spectrometric analysis. The CysPAT strategy was developed using a synthetic peptide, a standard protein and subsequently the strategy was applied to detect total Cys residuals from Hela cells lysate, achieving very high specificity and enrichment efficiency. The strategy was further developed to simultaneously enrich Cys peptides and phosphorylated peptides from SILAC labeled Hela cells subject to 5 min epidermal growth factor (EGF) stimulation, demonstrating high enrichment specificity for both PTMs and offering the possibility to detect free radical induced alteration in reversible Cys and to study the cross-talk between phosphorylation and reversible Cys modifications during cellular signaling.

## EXPERIMENTAL PROCEDURES

### 

#### 

##### Materials

All chemicals were purchased from Sigma-Aldrich (St. Louis, MO), unless otherwise stated. Modified trypsin was from Promega (Madison, WI). Poros R2 and Poros Oligo R3 reversed-phase material were from Applied Biosystems (Forster city, CA). GELoader tips were from Eppendorf (Hamburg, Germany). The 3 m Empore^TM^ C8 disk was from 3 m Bioanalytical Technologies (St. Paul, MN). Titanium dioxide beads were a gentle gift from GL Sciences Inc. (Tokyo, Japan). N-Succinimidyl Iodoacetate (SIA) was purchased from Thermo Scientific (Rockford, IL). Alkaline phosphatase, PNGase F and endoprotease Asp-N were obtained from New England Biolabs (Ipswich,MA). Glyko® Sialidase C™ was from Prozyme (Hayward, CA). All solutions were made with ultrapure Milli-Q water (Millipore, Bedford, MA).

##### Standard Peptide and Bovine Serum Albumin (BSA) Protein

The standard Cys peptide VALHCLAL was a generous gift of Prof. Peter Hojrup (University of Southern Denmark, DK), synthesized by solid phase peptide synthesis and dissolved in 50 mm triethylammonium bicarbonate buffer (TEAB). Bovine serum albumin (BSA) (0.5 mg) was dissolved in 200 μl 50 mm triethylammonium bicarbonate buffer (TEAB), pH 7.8, reduced with 10 mm TCEP for 1h at room temperature and digested with trypsin (1–2%, w/w) at 37 °C for 12 h, then desalted and purified with a self-packed R3 column for further use.

##### Cell Culture

Human cervix epithelial adenocarcinoma (HeLa) cells were cultured and maintained in 15 cm cell culture dishes in Dulbecco's Modified Eagle Medium (DMEM) + Glutamax media (Gibco, Waltham, MA) supplemented with 10% Fetal Bovine Serum (Sigma) and 1% penicillin/streptomycin (Invitrogen, Grand Island, NY). Cells were maintained at 37 °C in a humidified atmosphere containing 5% CO_2_. For the epidermal growth factor (EGF) stimulated stable isotope labeling using amino acids in cell culture (SILAC) HeLa cell experiment, HeLa cells were cultured for six cells divisions in DMEM SILAC light or heavy media supplemented with 10% FBS, 1% Glutamax and 1% penicillin/streptomycin and l-Met, l-Pro and Arg (Arg-0 or Arg-10) and Lys (Lys-0 or Lys-8). Prior to stimulation, cells were serum starved for 18 h at 80% confluence. Cells were stimulated with 150 ng ml-1 EGF in DMEM for 5 min. Then the cultures were washed with ice-cold phosphate buffer saline (PBS), followed by scraping the cells off the plate and pelleted by centrifugation. The remaining PBS was removed and the pellets were snap-frozen in liquid nitrogen. The pellets were stored at −80 °C until further sample preparation.

##### Chemical Synthesis of Cysteine-specific Phosphonate Adaptable Tag (CysPAT)

Firstly, for reagent solutions, 1 mg (3.53 μmol) SIA and 2 mg (15.99 μmol) 2-aminoethylphosphonic acid (2-AEP) was dissolved in 10 μl dimethyl sulfoxide (DMSO) and 150 μl 50 mm TEAB, respectively. The two reagent solutions were carefully mixed, adding the water-containing solution drop by drop to the DMSO solution and mixing. The final pH should be adjusted to 7.5 by adding 1 m TEAB, and the final volume was adjusted to 200 μl with 50 mm TEAB buffer. The reaction would happen immediately after mixing; if precipitation appears, it should be re-suspended by vortexing or pipetting up and down. The reaction is taken place in the dark for 1 h at room temperature to yield the CysPAT tag (2-(2-iodoacetamido) ethyl) phosphonic acid. The CysPAT tag should be freshly prepared before use and kept in the dark.

##### For Detection of Total Cys Residuals

HeLa cells derived from one plate were re-suspended in 500 μl lysis buffer containing 6 m urea, 2 m thiourea, 2% sodium dodecyl sulfate (SDS), complete protease inhibitor (Roche, Hvidovre, Denmark, one tablet per 50 ml), and lysed on ice using a probe sonicator and centrifuged for 10 min at 2000 × *g*. The supernatant was collected and subjected to 10 KDa spin filter to remove SDS. The precipitated protein pellet was redissolved in 100 μl urea-buffer (6 m Urea, 2 m Thiourea), and the proteins were reduced with 10 mm tris(2-carboxyethyl)phosphine (TCEP) for 1h at room temperature. No alkylation step was performed here. The reduced proteins were subsequently digested with 0.04 AU Lys-C (Wako, Japan) for 3 h at room temperature. After Lys-C digestion, the solution was diluted eight times with 50 mm TEAB buffer to 0.75 m urea and 0.25 m Thiourea, and trypsin (2%, w/w) was added for further digestion at 37 °C overnight. The digested peptides were desalted and purified with an OASIS HLB column (Waters, Millford, MA) and the eluted peptides were lyophilized and subsequently resuspended in 200 μl 50 mm TEAB buffer, and the concentration was measured using Qubit assay (Invitrogen). A total of 500 μg of peptides were used in the experiments. The peptides were further treated with 20 U of alkaline phosphatase, 1 μl PNGase F and 0.5 μl sialidase C at 37 °C for 3 h to perform the dephosphorylation and deglycosylation in order to avoid simultaneous enrichment of phosphorylated peptides and sialylated glycopeptides in the TiO_2_ enrichment step.

##### Preparation of Protein Lysate from EGF Stimulated SILAC Labeled HeLa Cells

The EGF stimulated SILAC Hela cells with light or heavy SILAC (EGF stimulated) labeling were re-suspended in 500 μl lysis buffer containing 6 m urea, 2 m thiourea, 2% SDS, 40 mm NEM, complete protease inhibitor (Roche, one tablet per 50 ml) and phosphatase inhibitor PhosStop (Roche, two tablets per 50 ml), and lysed on ice using a probe tip sonicator. After using 10 kDa spin filters to remove SDS and unreacted NEM, the protein concentration was determined by Qubit. A total of 250 μg of protein from light and heavy SILAC labeled Hela cells were pooled together and these samples were prepared in triplicate from three individual stimulation experiments. The following reduction, digestion were performed the same way as described for total Cys residuals, but without the dephosphorylation step.

##### Labeling of Cys Peptides with Synthesized CysPAT

For the standard peptide, 0.1 μg peptide was mixed with 5 molar ratio of the above prepared reaction buffer containing synthesized CysPAT. Peptides from 500 μg BSA, 500 μg HeLa cell protein extract for total Cys residuals and the coenrichment experiment, respectively, were mixed with 10 mm of the synthesized CysPAT in 50 mm TEAB, pH 7.8 in a volume of 200 μl. The mixed solution was incubated in the dark for 1 h with gentle agitation. Afterward, the solution was subjected to OASIS HLB column or Oligo R3 Reversed phase micro-column purification to purify the peptides and remove extra chemicals. The eluted peptides were lyophilized.

##### TiO_2_ Enrichment

TiO_2_ enrichment was performed to enrich the CysPAT labeled Cys peptides as previously described for phosphorylated peptides (20, 21). A total of 0.6 mg TiO_2_ per 100 μg peptides was used for total Cys peptides. For coenrichment of both reversible Cys modified peptides and phosphopeptides, a total of 1.4 mg TiO_2_ per 100 μg peptides was added to ensure efficient enrichment of both modified peptide sets.

The labeled peptides were dissolved in 100 μl 0.1% TFA, and then diluted 10 times with loading buffer of 80% ACN, 5% TFA and 1 m glycolic acid. Proper amount of TiO_2_ beads as described above was added to the solution, shaken for 10 min at 600 rpm and centrifuged. The supernatant was collected carefully and incubated with half the amount of TiO_2_. The TiO_2_ beads were first washed with 100 μl of loading buffer by mixing for 15 s, transferred to a new tube and centrifuged to pellet the beads, then washed with 100 μl of a solution containing 80% ACN and 1% TFA, and followed by washing with 100 μl of a solution containing 20% ACN and 0.1% TFA. The peptides were eluted with 50 μl eluting buffer (40 μl 28% ammonia solution in 960 μl water, pH 11.3) and centrifuged for 1 min. The eluent was collected and passed through a C8 stage tip to remove TiO_2_ beads and the peptides attached to the C8 tip were subsequently eluted with 1 μl 30% ACN. The enriched phosphorous peptides were acidified using formic acid, and desalted using a Poros Oligo R3 microcolumn. The desalted eluent was lyophilized prior to HILIC fractionation.

##### MALDI-TOF-MS

The standard peptide with or without phospho-tag labeling, and the labeled standard peptide spiked in normal BSA peptides before and after TiO2 enrichment were all desalted by reversed phase Poros Oligo R3 reversed-phase material staged in GELoader tips. Retained peptides were eluted onto a stainless-steel target plate using ACN/0.1% TFA (70:30, v/v) containing 5 mg/ml α-CHCA. Mass spectra were acquired on an Ultraflex II MALDI-TOF/TOF (tandem TOF) mass spectrometer controlled by flexControl software (version 2.4, Bruker Daltonics, Bremen, Germany). The instrument was operated in the positive reflector ion detection mode, and spectra were recorded in the mass range of *m*/*z* 500–4000. Typically, signals from 1000 laser shots (10 × 100 shots at 10 different positions) were averaged. Spectra were externally calibrated using a tryptic lactoglobulin. Mass spectra were analyzed with flex- Analysis software (version 2.4, Bruker Daltonics). For MALDI-TOF/TOF analysis, the window for precursor ion selection was set to be ±1% of the mass of the precursor ion.

##### Hydrophilic Interaction Liquid Interaction Chromatography (HILIC)

Enriched peptides from HeLa cells were further fractionated using HILIC. Enriched peptides were fractionated on an in-house packed TSKgel Amide-80 HILIC (Tosoh Bioscience, 5 μm) 320 μm × 170 mm μHPLC column by using the Agilent 1200 micro-HPLC instrument (26). Briefly, samples were suspended in solvent B (90% ACN, 0.1% TFA) by adding 10% TFA followed by water and finally the acetonitrile was slowly added to the aqueous solution in order to prevent peptide precipitation by the high acetonitrile concentration. Peptides were loaded onto a 320 ID peak HILIC column and eluted at 6 μl/min by decreasing the solvent B concentration (100–60% ACN, 0.1%TFAinwater) in 42 min. Fractions were automatically collected in a 96 well plate at 1 min intervals after UV detection at 210 nm and dried by vacuum centrifugation, and stored at −20 °C until LC-MS/MS analysis.

##### Mass Spectrometric Detection

Cysteine-containing peptides from BSA and HeLa cells for total Cys residuals were analyzed with a nanoEasy LC combined with a LTQ Orbitrap Velos mass spectrometer (Thermo Fisher Scientific). HILIC fractions were resuspended in 0.1% FA and loaded onto a 2 cm 100 ID pre-column using a nanoEasy LC (Thermo Fisher Scientific). Peptides were eluted directly onto the analytical column using a gradient of 0% to 34% buffer B (90% Acetonitrile, 0.1% FA) over 70 min. All LC-MS/MS runs were performed using an analytical column of 20 cm × 75 μm inner diameter fused silica, packed with C18 material (Dr. Maisch, Ammerbuch-Entringen, Germany). Mass spectrometry was performed using higher energy collision fragmentation (HCD) fragmentation on a Thermo LTQ Orbitrap Velos. MS settings: A full MS scan in the mass area of 400–1800 Da was performed in the Orbitrap with a resolution of 30,000 FWHM and a target value of 1 × 10^6^ ions. For each full scan the seven most intense ions (>+1 charge state) were selected for higher energy collision dissociation (HCD) and detected at a resolution of 7500 FWHM. The settings for the HCD were as follows: threshold for ion selection was 20,000, the target value of ions used for HCD was 1 × 10^5^, activation time was 10 ms, isolation window was 2.5 Da, and normalized collision energy was 34.

Reversibly modified Cys peptides and phosphopeptides from the simultaneous enrichment experiment were analyzed by an Orbitrap Fusion Tribrid™ Mass Spectrometer (Thermo Fisher Scientific). Peptides were resuspended in 5 μl of 0.1% TFA to their analysis. Peptides were loaded on an Acclaim PepMap100 C18 Nano-trap column (2 cm × 100 μm, 5 μm, 100 Å) using 0.1% TFA and separated on an in-house packed Reprosil-Pur C18-AQ (3 μm; Dr. Maisch GmbH) analytical column (20 cm × 75 μm) at using a Dionex Ultimate® 3000 Nano LC system (Thermo Scientific) and eluted at a flow of 250 nL/min. Mobile phase was acetonitrile (B) and water (A) both containing 0.1% formic acid. Depending on the samples, gradient was 0–34% solvent B in 90 min, 34–100% B in 5 min, and 8 min at 100% B. Eluting peptides were analyzed on an Orbitrap Fusion. Survey scans of peptide precursors from 350 to 1400 *m*/*z* were performed at 120,000 resolution (at 200 *m*/*z*) with a 5 × 10e5 ion count target, the max injection time was 60 ms. Tandem MS was performed by isolation at 1.6 Th with the quadrupole, HCD fragmentation with normalized collision energy of 38, and MS2 analysis in the Orbitrap. The MS2 resolution was set to 30,000 and the max injection time was 100 ms. Only those precursors with charge state +2, +3, and +4 were sampled for MS2. The dynamic exclusion duration was set to 30 s with a 10 ppm tolerance around the selected precursor. The instrument was run in top speed mode with 3 s cycles, meaning the instrument would continuously perform MS2 events until the list of nonexcluded precursors diminishes to zero or 3 s, whichever is shorter. Raw data were submitted to pride (http://www.ebi.ac.uk/pride/archive/) under the project accession PXD002704 (username: reviewer18039@ebi.ac.uk, password: t5mWGpOb).

##### Bioinformatics

The LC-MS/MS data were processed with Proteome Discoverer (Version 1.4.1.14, Thermo Fisher Scientific) and subjected to database searching using both an in-house Mascot server (Version 2.2.04, Matrix Science Ltd., London, UK) and the embedded Sequest HT server with the following criteria: database, SwissProt Homo Sapiens protein database (updated 15–01-2015, 20193 sequences) with common contaminants; enzyme, trypsin; maximum missed cleavages, 2; variable modifications included oxidation (Met), acetyl (protein N terminus and lysine(K)), SIA for Cys (SIA represented the CysPAT tag), deamidation for Asn and Gln. For the coenrichment experiments, only Mascot was used as search engine, and beside above parameters, NEM for Cys, phosphorylation (Ser, Thr, and Tyr), SILAC (Arg10/Lys8) were also included as a variable modification. The MS and MS/MS results were searched with a precursor mass tolerance at 10 ppm and a MS/MS mass tolerance at 0.05 Da. The Mascot results were filtered in Proteome Discoverer with the integrated Percolator algorithm (27) to ensure the false discovery rate (FDR) less than 0.01. Only peptides identified with high confidence, Mascot score higher than 18 and passed the default score *versus* charge state for Sequest HT were accepted. Peptides with different amino acid sequences or modifications were considered unique. The quantitative data from the triplicate SILAC labeled HeLa cells subjected to EGF stimulation was submitted to statistical analysis. The log2-values of the measured precursor areas were normalized by the median. Phosphosite localization probability was determined using the PhosphoRS probability algorithm (28). Peptides were merged with the R Rollup function (http://www.omics.pnl.gov) allowing for one-hit-wonders and using the mean of the normalized areas for each peptide. Then the mean over the experimental conditions for each peptide in each replicate was subtracted in order to merge data from the three replicates. Significant up/down regulated peptides were defined as peptides with minimum 1.5-fold change, quantified in at least two of the replicates and with a standard deviation lower than the 2× median of all the standard deviations of peptides. Annotation and classification of the identified proteins were facilitated by using Protein Center (Thermo Fisher Scientific) and the PANTHER Classification System (http://www.pantherdb.org/). The detail procedures were performed as described previously (29). Ingenuity pathway analysis (IPA) was performed to reveal the protein interaction and signaling pathways.

## RESULTS AND DISCUSSION

### 

#### 

##### Development of the CysPAT Chemistry and Test of Cys Labeling

**Fig. 1.**
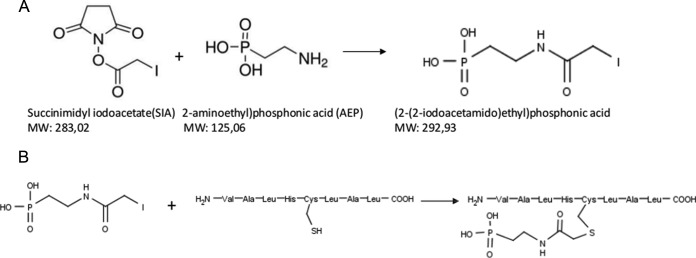
**Chemical synthesis and peptide labeling using the CysPAT tag.** *A*, Synthesis of 2-(2-iodoacetamido) ethyl phosphonic acid (CysPAT) by reaction between N-Succinimidyl iodoacetate (SIA) and 2-aminoethylphosphonic acid (2-AEP) at 1:5 molar ratio at pH = 7.8. *B*, Cys peptide labeling was performed through reaction between the free thiol group and the iodo group of CysPAT.

In this manuscript, a Cysteine-specific Phosphonate Adaptable Tag (CysPAT) was synthesized by a reaction between N-Succinimidyl iodoacetate (SIA) and 2-aminoethylphosphonic acid (2-AEP), in a 1:4 molar ratio, in a DMSO/TEAB (pH = 7.8) solution for 1 h at room temperature to generate 2-(2-iodoacetamido) ethyl phosphonic acid. Fig. 1*A* depicts the chemical scheme for the synthesis. Succinimidyl iodoacetate (SIA) was selected as it is a small heterobifunctional crosslinker with sulfhydryl- and amine-reactivity through its iodoacetate- and succinimidyl moiety, respectively (Fig. 1*A*). Apart from SIA, we also tested other similar molecules, such as succinimidyl-(4-iodoacetyl)-aminobenzoate as a viable option for thiol tagging; however SIA showed the highest labeling performance (data not shown). The SIA molecule allowed the introduction of a phosphonate functional group on the Cys residue via alkylation (Fig. 1*B*), which could be used for subsequent enrichment using TiO_2_, a chromatographic resin with high efficiency for enrichment of phosphorylated peptides in phosphoproteomics.

**Fig. 2.**
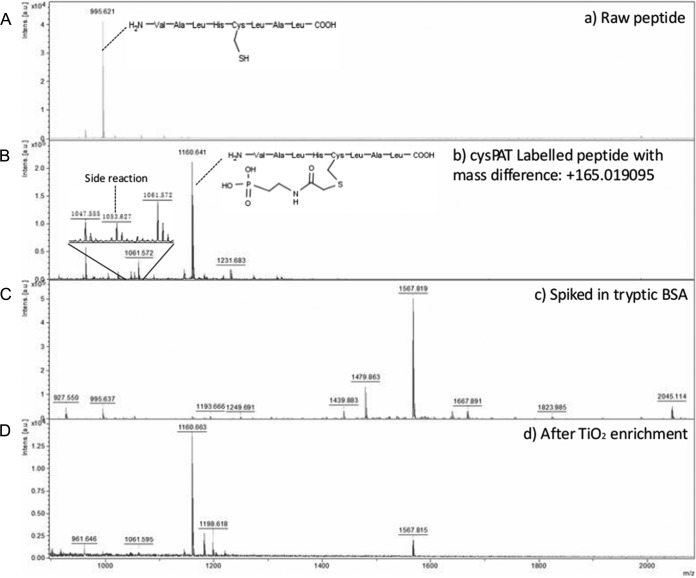
**CysPAT labeling allows a highly selective enrichment of cysteine-containing peptide using TiO_2_.** MALDI-MS spectra of the *A,* standard synthetic Cys peptide; *B*, CysPAT-labeled peptide. Zoom in the 1045–1065 m/z range shows the presence of a possible side reaction product with less than 1% intensity; *C*, CysPAT-labeled peptide spiked in tryptic digest of BSA in a 1:1000 ratio, and *D*, enriched CysPAT-labeled peptide by TiO_2_ from tryptic BSA mixture.

The CysPAT enrichment strategy was developed using a synthetic Cys peptide (VALRHCLAL, *m*/*z* 995.58). The CysPAT tag was incubated in a 5:1 molar ratio with the synthetic peptide at room temperature for 1h. The iodoacetamide group specifically reacts with the free thiol group of the Cys residue through alkylation. As monitored by MALDI MS the alkylation between the CysPAT tag and the peptide was highly efficient and led to a 165.02 Da mass increase compared with the unreacted standard Cys peptide (Fig. 2*A* and 2*B*). MALDI-MS/MS confirmed that the modification occurred at the Cys residue (supplemental Fig. S1*A*). Less than 1% of the standard peptide reacted with residual iodoacetic acid, formed by the hydrolysis of the SIA compound (insert Fig. 2*B*), illustrating the efficiency of the tagging strategy.

Next the CysPAT-labeled peptide was spiked into a peptide mixture originating from tryptic digestion of reduced and iodoacetamide-alkylated bovine serum albumin (BSA) in a 1000 molar excess (see MALDI MS analysis of the mixture in Fig. 2*C*). The Cys-PAT peptide was subsequently enriched using TiO_2_ chromatography, in a similar way as for phosphopeptides (20) (Fig. 2*D*). In the spectrum of the spiked BSA peptides mixture (Fig. 2*C*), most of the intense peaks are from known BSA peptides, and the signal of the CysPAT-labeled peptide is suppressed. However, after TiO_2_ enrichment, the signal of the CysPAT-labeled peptide was the most intense compared with weak signals from some BSA peptides (Fig. 2*D*). These results indicated that the CysPAT-labeled Cys peptide can be efficiently enriched using TiO_2_ chromatography with high efficiency and specificity.

Next, the method was applied to peptides derived from BSA. BSA is a protein containing 35 Cys residues among a total of 607 amino acids and 34 are in disulfide bridges. After reduction, alkylation with CysPAT and trypsin digestion, the peptide mixture was subjected to TiO_2_ enrichment and the enriched peptides were further analyzed by nLC-MS/MS. Subsequent database search revealed that all the Cys peptides were identified with high confidence (1% FDR) (supplemental Fig. S2). A representative MS/MS spectrum of a CysPAT-labeled peptide from BSA is reported in supplemental Fig. S1*B*. No neutral loss was observed in the fragment ions because of the gas phase stability of phosphonate functional group. Interestingly, a diagnostic ion at 224.014 m/z (C_6_H_11_NO_4_PS) was present in all the MS/MS spectra of CysPAT-labeled peptides. A possible structure of the diagnostic ion is reported in supplemental Fig. S1*C*.

In order to characterize the chromatographic behavior of the CysPAT tagged peptides we made a peptide mixture containing equal amount of CysPAT and iodoacetamide (IAA)-labeled Cys peptides from BSA. Characterization of the chromatographic behavior of this peptide mixture revealed that the addition of an anionic phosphonate moiety by CysPAT increased the retention time compared with IAA-labeled Cys peptides in C18 reversed-phase (RP) chromatographic separation (supplemental Fig. S3*A* and S3*B*). The chromatographic shift was linearly dependent on the number of modified Cys (supplemental Fig. S3*B*). In addition, the phosphonate moiety slightly reduced the peptide charge state but did not give raise to gas-phase beta elimination during CID or HCD fragmentation, a phenomenon commonly observed with the phosphate moiety when fragmenting serine and threonine phosphorylated peptides, thereby facilitating accurate identification of the SIA modified peptide and the site for modification. Furthermore, because of the phosphonate moiety the SIA labeled peptides are resistant toward phosphatase treatment enabling a clear distinction between phosphorylated peptides and SIA labeled peptides if needed.

##### Analysis of Cys Peptides from HeLa Cells

**Fig. 3.**
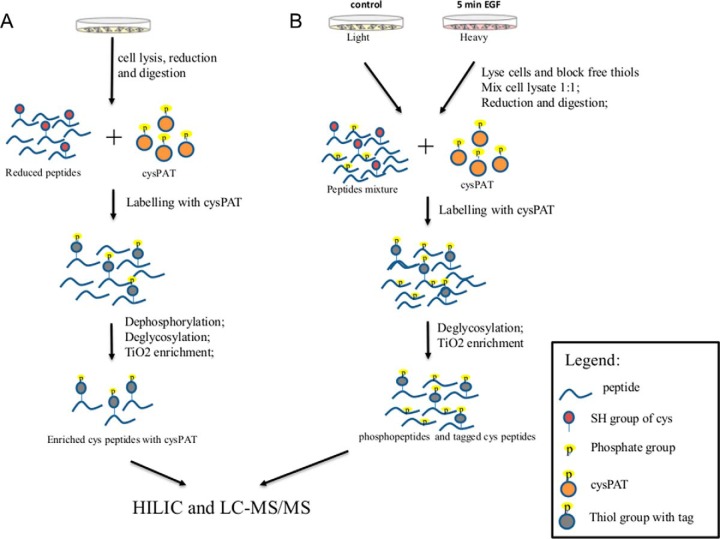
**General workflow for CysPAT labeling of complex biological samples.** *A*, Selective detection of total Cys peptides from Hela cells. *B*, Coenrichment of reversibly modified Cys peptides and phosphopeptides from SILAC labeled Hela cell subject to 5 min EGF stimulation. All labeled peptides were enriched with TiO_2_ before HILIC prefractionation and nLC-MS/MS analysis.

The CysPAT strategy was next applied to detect total Cys residuals (including both free and reversibly modified Cys) from 500 μg HeLa cell lysates under physiological condition (Fig. 3*A*). The proteins were reduced with TCEP, tryptic digested and alkylated with the CysPAT tag. Prior to TiO_2_ enrichment the sample was dephosphorylated and deglycosylated as both phosphopeptides and sialylated glycopeptides have a high affinity toward the TiO_2_ resin (20, 30). The CysPAT tagged peptides were enriched with TiO_2_, fractionated with hydrophilic interaction liquid chromatography (HILIC), and analyzed with LC-MS/MS. In total, 7628 unique total peptides were identified of which 7509 unique Cys peptides (6108 unique Cys sites) were originating from 2750 proteins (supplemental Table S1). The enrichment efficiency was 98.4%. In a similar experiment where the free Cys were blocked with NEM and the reversibly modified Cys were labeled with CysPAT, a total of 5687 unique Cys peptides were identified from 500 μg of HeLa cells lysate (supplemental Table S2).

##### Strategy for Simultaneous Enrichment of Cys-containing Peptides and Phosphopeptides Applied to Study the Effect of 5 min EGF Stimulation of HeLa Cells

As TiO_2_ chromatography is widely used for efficient enrichment of phosphopeptides, we next tested if it was possible to perform a simultaneous enrichment of phosphopeptides and reversibly modified CysPAT labeled peptides by TiO_2_ chromatography. Such a strategy would allow an investigation of the potential cross-talk between phosphorylation and Cys modification from the same sample. Therefore, we designed an experiment for simultaneous enrichment of phosphopeptides and reversibly modified CysPAT labeled peptides from SILAC labeled Hela cells subjected to 5 min EGF stimulation (see strategy in Fig. 3*B*). Here, the free Cys residues were firstly blocked with NEM in the cell lysis buffer, then the differentially SILAC labeled proteins were purified, quantified and equally mixed (250 μg from each cell population). The mixed protein mixture was reduced with TCEP, tryptic digested, labeled with CysPAT, and deglycosylated with PNGase F and Sialidase A. More TiO_2_ beads than for phosphopeptides were used for enrichment in order to achieve efficient enrichment of both types of modified peptides (31). The enriched peptides were fractionated into 14 fractions using HILIC and analyzed using the Orbitrap Fusion mass spectrometer.

**Fig. 4.**
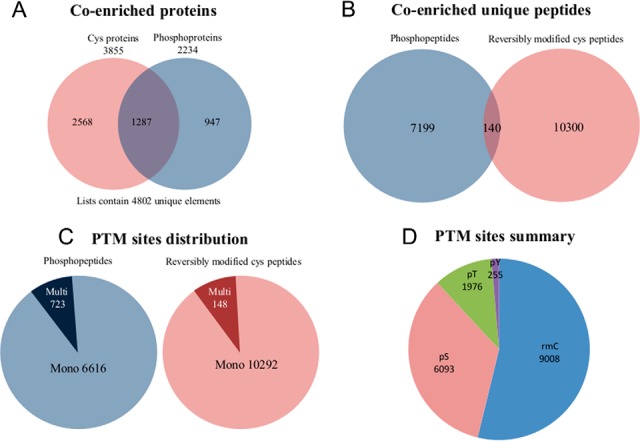
**CysPAT strategy allows high efficient coenrichment of Cys peptides and phosphorylated peptides.** Venn diagram indicated *A,* the identified proteins and *B,* the unique peptides with or without reversible Cys modification and phosphorylation from the coenrichment experiment. *C,* the summary of mapped mono and multi phosphorylation sites and reversibly modified Cys sites, *D*, PTMs distribution.

As expected, both phosphopeptides and CysPAT modified peptides were simultaneously enriched with the TiO_2_ resin. In total, 4802 proteins were identified in this study; they included 3855 proteins containing CysPAT labeled Cys peptides and 2234 proteins identified with phosphopeptides, with 1287 proteins shared between the two groups (Fig. 4*A*). At the peptide level, we identified a total of 20408 unique peptides within the three biological replicates, which include 7339 unique phosphopeptides and 10440 unique CysPAT peptides (Fig. 4*B*, supplemental Table S3). A total of 2769 peptides did not contain the CysPAT and/or phosphorylation modification, giving enrichment efficiency for both types of modified peptides of 87%. A total of 140 peptides were found to contain both phosphorylation sites and reversibly modified Cys sites. This low overlap between phosphopeptides and reversibly modified Cys peptides is most likely reflecting the fact that phosphorylation happens through an enzymatic reaction by kinases, which mostly recognize amino acids on the surface of the proteins, whereas reversible Cys modifications are mostly located inside the protein 3-D structure, such as disulfide bonds, which are the main component of reversible Cys modifications. Thus, phosphorylation sites and reversible modified Cys are not in close proximity in the protein sequence. The enriched peptides also included 723 peptides with multiple phosphorylation sites and 148 with multiple CysPAT sites (Fig. 4*C*). Based on the identified peptides, 8324 unique phosphorylation sites (6093 pS, 1976 pT, 255 pY) and 9008 unique reversibly modified Cys sites (Fig. 4*D*) were successfully mapped, respectively. The information of the identified unique Cys sites and phosphorylation sites retrieved from Uniprot database is presented in supplemental Table S4. Furthermore, phosphopeptides and CysPAT peptides showed diverse elution patterns during the HILIC fractionation step (supplemental Fig. S4). Earlier elution of CysPAT labeled Cys peptides was observed in the HILIC fractionation compared with phosphorylated peptides, indicating that the CysPAT labeled Cys peptides are generally more hydrophobic than the phosphopeptides. This could be attributed to the alkyl chain moiety of the tag that confers more hydrophobicity. This different property of phosphopeptides and CysPAT labeled peptides enables efficient prefractionation using HILIC chromatography to increase the identification efficiency while decrease the interference and competition between both types of peptide subsets during MS characterization in coenrichment experiments. These results confirmed that the phosphopeptides and the Cys peptides labeled with CysPAT can be efficiently simultaneously enriched by a routine TiO_2_ enrichment method with high enrichment efficiency, and HILIC prefractionation can partially fractionate these two types of peptides because of their different hydrophilicity.

According to the Uniprot database some of the reversibly modified Cys sites were confirmed key sites for zinc finger structure (39 sites in 32 proteins), iron-sulfur cluster (18 sites in 14 proteins), S-nitrosylation (16 sites from 13 proteins) and other important functionalities. The oxidation status of the conserved Cys residues in these proteins determines the protein structure and activity (32, 33). The annotated PTM sites information from Uniprot database was presented in supplemental Table S4.

##### Regulation of Phosphorylation and Reversible Cys Modification During Short Time EGF Stimulation of HeLa Cells

##### Overview of the Analysis

Extracellular EGF stimulation directly leads to the activation of the epidermal growth factor receptor (EGFR) through triggering the dimerization and autophosphorylation on key cytoplasmic tyrosine residues of EGFR (34). It further activated a multitude of downstream proteins and initiates a cascade of signaling events. These processes have previously been shown to be regulated by multiple PTM events, such as phosphorylation, ubiquitination and acetylation (35). Studies revealed that short term EGF stimulation (5 min) can lead to the sharply increased phosphorylation of EGFR and multiple downstream proteins whereas the total protein amounts were stable (36, 37). Meanwhile, short term EGF stimulation can result in a transient increase in the intracellular concentration of H_2_O_2_ (38), which may induce the intracellular oxidative levels, and increase reversible Cys oxidation. Phosphorylation of EGFR and H_2_O_2_ production both reach to highest levels at 5 min in time courses of EGF stimulation (37, 38), therefore, we applied the coenrichment method to simultaneously determine the changes of phosphorylation and reversible Cys oxidation in SILAC labeled Hela cells subjected to 5 min EGF stimulation (Fig. 3*B*).

A total of 2255 peptides from 1313 proteins (supplemental Table S5) were considered as significantly regulated peptides, which should be quantified in at least two out of three replicates, with more than 1.5-fold change (log2(H/L) ≥ 0.59 or ≤ −0.59), and with a standard deviation lower than the median of the global peptide standard deviation. The summary of down and up-regulated Cys peptides and phosphopeptides as well as related proteins are presented in supplemental Fig. S5. Surprisingly, we identify more regulated Cys peptides compared with phosphorylated peptides in this study, indicating a substantial modulation of cellular signaling through reversible Cys modifications. Most of Cys in cells exist in a balanced condition between free form and reversibly oxidized form. In this study, we blocked the free thiols, reduce and enrich the reversibly modified Cys peptides, so the identified down-regulated Cys peptides indicated that these sites were present more as free thiols, conversely, the identified up-regulated Cys peptides indicated that these sites were more reversibly oxidized. Three examples of MS/MS spectra from MCM3, PTP1B and Filamin A are reported in supplemental Figs. S6, S7, and S8, respectively. As it was only 5 min EGF stimulation, we assumed that the total protein expression should not be significantly affected.

##### The EGFR Network

Our study identified 8 reversibly modified Cys sites and 3 phosphorylation sites from EGFR itself. Among these, significant regulation was observed for Cys157, Cys260, Cys291, Cys307, Cys311 within the extracellular part of EGFR. The Cys sites were all down-regulated in their reversibly modified form and they are all located in the cysteine rich domain II or S1of EGFR and form disulfide bonds with other Cys in the same domain (39). Domain II contain the dimerization loop which is essential for the dimerization of EGFR upon EGF stimulation, and in normal physiological conditions the autoinhibitory intramolecular domain II-IV interactions can maintain the EGF receptor in an inactive state by constraining the relative orientation of ligand binding domains I and III (40). The reversible Cys modification observed on EGFR could be a result of the activation of the protein disulfide-isomerase, which can act as a reductase the cell surface, on which we identified significant modulation of Cys312 and Cys343. The Cys797 in the EGFR active site, which has previously been shown to be regulated by H_2_O_2_ (41), was not identified in the present study. The intracellular autophosphorylation site Tyr1172 was confirmed to be phosphorylated at very high level upon 5 min EGF stimulation verifying the autophosphorylation and EGF stimulation (37).

**Fig. 5.**
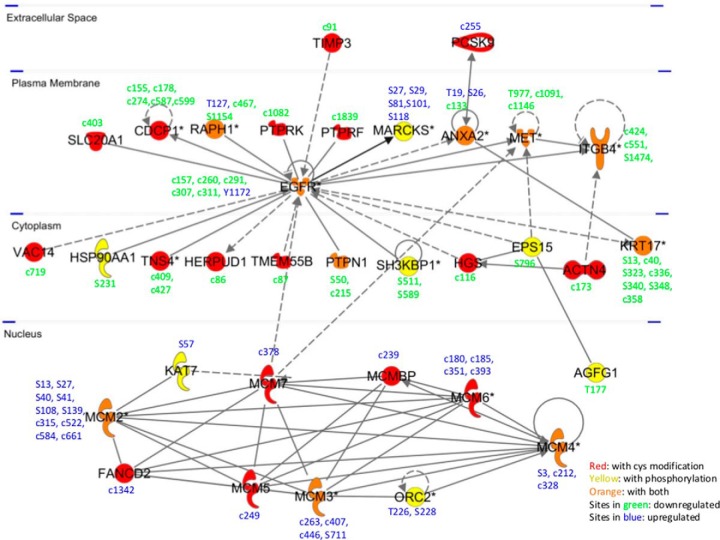
**The regulated PTM sites and related proteins in the EGFR interaction network.** The regulated PTM sites together with their change pattern and protein cellular localization were indicated. For proteins marked in red: with only Cys modification; in yellow: with only phosphorylation; in orange: with both PTMs. For PTM Sites in green: downregulated; in blue: up-regulated.

Protein interaction analysis of the regulated proteins revealed that 21 proteins interacted directly with the EGFR, and 12 proteins showed indirect interaction, as shown in Fig. 5. The regulated phosphorylation sites and reversibly modified Cys sites together with their regulation and protein cellular localization are indicated in the figure. The analysis showed that many of the phosphorylation sites and reversibly modified Cys sites from these proteins were significantly regulated by 5 min EGF stimulation. EGFR and 9 other EGFR network proteins were determined to have both regulated phosphorylation sites and Cys sites, and some of these sites showed reverse regulation patterns within the same protein, such as for EGFR, Ras-associated and pleckstrin homology domains-containing protein 1 (RAPH1) and Annexin A2 (ANXA2) (supplemental Table S6). The minichromosome maintenance (MCM) protein complex located in the nucleus is required for initiation and regulation of DNA replication and cell proliferation, specifically the formation and elongation of the replication fork (42). This protein complex is part of the EGFR network. For example, it has previously been shown that activated EGFR can enhance MCM7 phosphorylation and subsequently DNA replication (43). Here, we identified 10 molecules forming a MCM complex network, including all six members of the MCM hexamer (MCM2 through MCM7) which form a ring structure (44). We found that all the changed Cys sites and phosphorylation sites of the 10 proteins were up-regulated, indicating increased phosphorylation and reversible Cys oxidation of MCM complex upon 5 min EGF stimulation, however, the exact biological role of these sites is unknown.

**Fig. 6.**
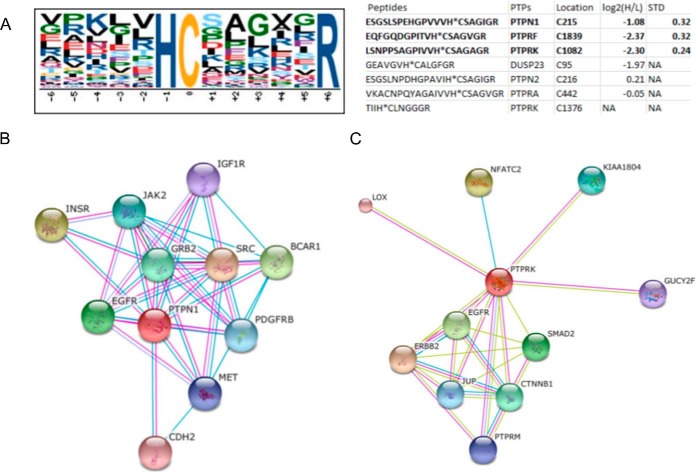
**HC(X)5R motif logo and representative protein-protein interaction networks.** *A,* Logo of identified putative phosphatase signature motif HCX5R and related peptides from PTPs as well as their quantification information, the peptides in bold size were significantly regulated, NA, not available. *B,* STRING protein-protein interaction network of the PTPN1 and PTPRK phosphatases.

Another integrated part of the EGFR network is the Protein tyrosine phosphatases (PTPs) that catalyze the dephosphorylation of phosphotyrosine residues. These enzymes are key regulators in signal transduction pathways mediated by tyrosine phosphorylation, and are involved in the control of cell growth, proliferation, differentiation, and transformation (45). The PTPs carry the highly conserved active site motif HC(X)5R (PTP signature motif), where the histidine and Cys residues are conserved in PTPs and dual-specific phosphatases (DUSPs) (46). The HC(X)5R motif is essential for the catalytic activity of PTPs. PTPs use the free thiol group of the active Cys site within this motif as the attacking nucleophile to form a cysteinyl-phosphate enzyme intermediate (47). Mutagenesis-directed substitution or oxidation of the Cys residues would completely abolish PTPs activity (48). In the present study, the HC(X)5R motif (Fig. 6*A*) was identified in 36 of our identified unique Cys peptides from 35 proteins (Supplemental Table S7), including 6 protein phosphatases, PTPN1/PTP1B, PTPN2, receptor-like PTPs (PTPRF, PTPRK, PTPRA), and DUSP23. The reversible form of catalytic Cys sites identified in PTP1B, PTPRF, and PTPRK were all significantly down-regulated, indicating they were reduced and these PTPs were activated. These PTPs were also found in the interaction network of EGFR (Fig. 6*B* and 6*C*), were PTP1B, PTPN2 and receptor-like PTPs are known to be negative regulators of the phosphorylation of EGFR, as summarized and further confirmed by Tarcic *et al.* (49). In addition, we identified regulation of Cys49 in Protein tyrosine phosphatase type IVA 1, which is known to form disulfide bridges with Cys104 (the phosphocysteine intermediate). This phosphatase is strongly associated with the ras homolog family members RAC1, RHOC, and RHOA and with ubiquitin (supplemental Fig. S9).

The results indicated that the reduction of catalytic Cys sites of PTPs would lead to the activation of PTPs within 5 min EGF stimulation, which should contribute to the dephosphorylation of EGFR and other proteins in downstream signaling events of EGFR. The EGF stimulation increase the intracellular production of ROS, such as H_2_O_2_, However, as the catalytic Cys sites of PTPs were reduced in the stimulation process, indicating an activation of the PTPs and at the same time we observed activation of the pathway. These findings could reflect the fact that we perform a continuous stimulation of the EGFR where EGF is present all the time. This means that we will have a constant autophosphorylation of EGFRs and most likely an increased dephosphorylation at the same time via the activated PTPs. It would be very interesting to explore the mechanisms underlying the reduction of the Cys in the active site of PTPs within the cell with and without constant EGF stimulation.

PTP1B is a well characterized phosphatase involved in the regulation of the insulin receptor and EGFR and a therapeutic target in type 2 diabetes and obesity (50, 51). Here, we identified 2 reversibly modified Cys sites (Cys 32 and Cys215) and 5 phosphorylation sites (Ser50, Ser295, Ser363, Ser365, and Ser378) in PTP1B. Oxidation of the Cys215 site in the HC(X)5R motif would lead to the inactivation of PTP1B, however, the reversible sulfenylation of Cys215 is a protective intermediate in the oxidative inhibition of PTP1B, it can protect the Cys from further irreversible oxidation and may facilitate reactivation of PTP1B by biological thiols (52). The activity of PTP1B has also been shown to be allosteric regulated by phosphorylation of the Ser50 residue which results in the inactivation of this protein (53). Our results showed both the reversibly modified form of Cys215 and the phosphorylation of Ser50 was downregulated, thereby the free form of Cys 215 should be increased, and the inhibitory effect of phosphorylation of the Ser50 would be decreased, both events would contribute to the activation of PTP1B.

Further in the well-known EGFR signaling pathway we observed regulated phosphorylation sites or Cys sites in four more proteins (supplemental Table S5, supplemental Fig. S10), Inositol 1,4,5-trisphosphate receptor type 3 (ITPR3) and Ras GTPase-activating protein 1(RASA1) with down-regulated Cys sites, mitogen-activated protein kinases (MAPK1 and MAPK3) with up-regulated phosphorylation sites. RASA1, MAPK1 and MAPK3 are the key molecules in the EGFR-Ras-Raf-MEK-ERK signaling network, which has been the subject of intense research and pharmaceutical scrutiny to identify novel target-based approaches for cancer treatment (54). The Thr185, Tyr187 of MAPK1 and Thr202, Tyr204 of MAPK3 are key phosphorylation sites of the Thr-Xaa-Tyr motif in the MAPK activation loop, MAPKs are activated by phosphorylation of these two specific amino acids in the activation loop, and dephosphorylation of the tyrosine residue is sufficient to inactivate MAPK, blocking its nuclear translocation (55). The phosphorylation Thr185, Tyr187 of MAPK1 and Thr202, Tyr204 of MAPK3 were all identified to be up-regulated upon 5 min EGF stimulation, which is consistent with previous similar phosphoproteomic study (56). The inactivation of MAPKs can be achieved by PTPs, such as MAPK-PTPs and DUSPs (55). In particular, DUSP23 can dephosphorylate and inactivate MAPK3 (57). In this study we found the catalytic Cys95 of DUSP23 to be more in the free thiol form, indicating that DUSP23 might also be activated and dephosphorylate the downstream targets, like MAPK3.

##### Global Pathway Analysis of Regulated Reversible Cys Proteins

**Fig. 7.**
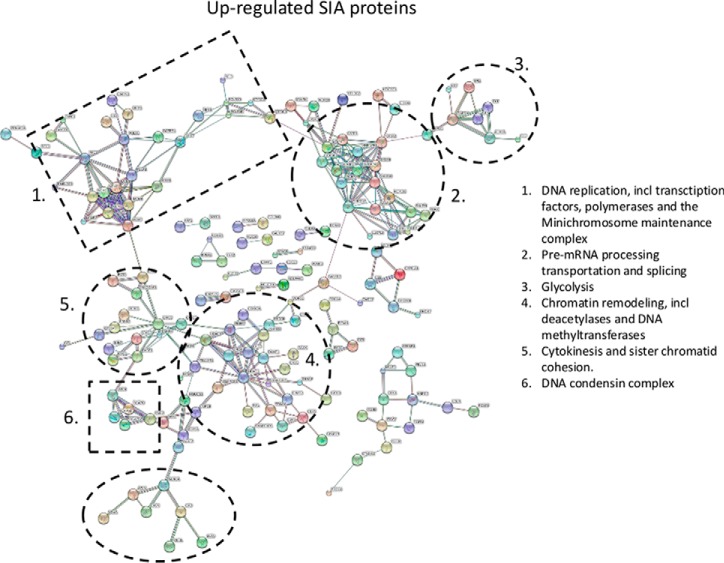
**Protein-protein interaction network of proteins identified with up-regulated reversibly oxidized cysteine residues SILAC-ratios upon EGF stimulation of Hela cells.** Several biological processes were identified such as: (1) DNA replication (transcription factors, polymerases and the Minichromosome maintenance complex); (2) Pre-mRNA processing transportation and splicing; (3) Glycolysis; (4) Chromatin remodeling, (deacetylases and DNA methyltransferases); (5) Cytokinesis and sister chromatid cohesion and 6) DNA condensin complex.

**Fig. 8.**
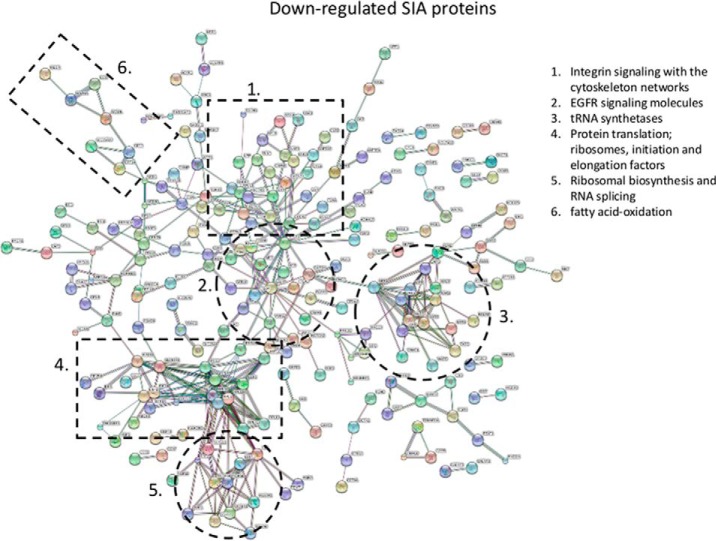
**Protein-protein interaction network of proteins identified with downregulated reversibly oxidized cysteine residues SILAC-ratios upon EGF stimulation of Hela cells.** (1) Integrin signaling with the cytoskeleton networks; (2) EGFR signaling molecules; (3) tRNA synthetases; (4) Protein translation; ribosomes, initiation and elongation factors; (5) Ribosomal biosynthesis and RNA splicing; (6) Fatty acid-oxidation.

A total of 836 proteins were found to include significant alteration in the oxidation state of Cys sites. Global STRING analysis of all up- and down-regulated proteins can be visualized in Fig. 7 and 8, respectively. From this analysis it is apparent that a substantial amount of proteins in cellular networks are modulated by reversible Cys modifications upon 5 min EGF stimulation, most likely because of the increased generation of H_2_O_2_ observed after 5 min stimulation (38). Many of the proteins have never previously been associated with EGF signaling or reported to be regulated by reversible Cys modifications. Brief EGF stimulation of HeLa cells not only result in regulation of proximal cellular events, such as the EGF signaling pathway itself and integrin signaling, but also significant regulation of molecular events within the nucleus, such as chromatin remodeling, DNA condensation, initiation of cytokinesis and modulation of pre-RNA (Fig. 7 and 8). Interestingly, among the chromatin remodeling cluster observed with increased SIA modification we identify two HDAC proteins, HDAC 1 and 3, which are key deacetylases for deacetylation of lysine residues on the N-terminal part of the core histones (H2A, H2B, H3, and H4). Both Cys sites identified in HDAC 1 (Cys273) and HDAC 3 (Cys279) was identified as up-regulated twofold after 5 min EGF stimulation. It has previously been shown that oxidative reversible modification of two conserved cysteine residues, corresponding to Cys(261) and Cys(273) in HDAC1, coincided with attenuation of histone deacetylase activity, resulting in subsequent changes in histone H3 and H4 acetylation patterns (58) and altered gene expression regulation. Our data strongly suggest regulation of deacetylation activity of HDAC 1 and 3 after brief EGF stimulation.

In addition, we identified 61 Cys sites on 40 proteins from the mitochondria that show alteration in reversible Cys modification. These proteins are involved in the electron transport chain and oxidative phosphorylation and in protection against oxidative stress (*e.g.* Persulfide dioxygenase ETHE1 and Phospholipid hydroperoxide glutathione peroxidase, (supplemental Fig. S11).

Furthermore, we identified a larger number of proteins, with regulated reversible Cys modifications, that are involved in general redox regulation in the cell, as illustrated by the STRING network in supplemental Fig. S12. Of these, thioredoxin was identified with one significantly down-regulated Cys site (Cys73). This site has previously been shown to be nitrosylated (59) and it can function as donor for nitrosylation of target proteins, such as Caspase-3, which lead to inhibition of apoptosis (59).

Some of the most abundant protein networks observed in the down-regulated reversible modified Cys data set are related with protein translation, such as ribosomal proteins, translation initiation and elongation factors and tRNA synthetases (Fig. 8). Surprisingly, a total of 34 Cys sites on 13 tRNA synthetases showed differential regulation, 32 down regulated, in their Cys site, after brief EGF stimulation. Aminoacyl-tRNA synthetases catalyze the process of aminoacylation of tRNAs by joining an amino acid to its correct tRNA, that can then be used in the protein translation machinery. The large amount of regulated tRNA synthetases suggests that Cys modulation is a common and general way of regulating the activity of this enzyme group in mammalian cells. A few previous studies of tRNA synthetases from prokaryotes have suggested a role for cysteine residues in the aminoacylation process (60). In one of the most previous study they found that air oxidation increased the Ser-tRNA^Thr^ level in the presence of elongation factor Tu and that Cys182 forms a putative metal binding site with three conserved histidine residues (His73, His77, and His186) (61). However, because the content of cysteine in general is higher in the mammalian tRNA synthetases compared with the prokaryote ones, we propose that reversible Cys modification of tRNA synthetases via oxidative species, such as H_2_O_2_, is the key regulator of the aminoacylation process in eukaryote systems and especially in conjunction with cellular signaling where new protein has to be generated fast.

Previously, reversible Cys modification has been proposed to be involved in cross talking with various other PTMs, such as phosphorylation, acetylation and ubiquitination (62). However, beside the abovementioned tyrosine-phosphatases, very little evidence of this cross talking in cellular signaling has been presented. In the present study, we observed alteration in Cys modifications of several enzyme groups responsible for the addition and removal of post-translational modifications, such as for the phosphatases described above. Among these, we identified a total of 30 kinases that showed alteration in reversible Cys modification, including GSK3A, PRKAR1A, MAP2K2, DYRK1B, ERBB2, ARAF, and SLK, further supporting the idea of a substantial cross-talking between Cys modification and phosphorylation. Furthermore, we observed significant alteration of reversible Cys modification of enzymes participating in Acetylation (HDAC1 and 3, KAT6B, KAT7, and HAT1), O-GlcNAcylation (OGT), Neddylation and Ubiquitination (see supplemental Fig. S13) and Glycosylation (GALB1, MGEA5, GALNT2, STT3A, ALG9). These results suggest that the reversible Cys modification is cross-talking with a substantial amount of PTMs during brief cellular stimulation, placing this modification as a central part of cellular signaling, at a level we previously did not anticipate. However, the exact way this cross talk is conducted is still relative unknown unless for the enzyme where a direct activity switch (tyrosine phosphatases) or a metal conjunction is observed which include a free Cys residue.

## CONCLUSION

In this study, we have developed a new phosphonate tag capable of tagging cysteine containing peptides and demonstrated with standard peptides and complex mixtures that the strategy has a very high efficiency and specificity. In addition, because of the high affinity of the phosphonate tag and phosphate groups for TiO_2_, the new tagging of Cys peptides allow the simultaneous enrichment of phosphopeptides and Cys peptides which offers an excellent tool for studying the cross-talk between phosphorylation and Cys redox modifications. In principle, other enrichment methods developed for phosphopeptides should also be applicable to enrich the Cys peptides labeled with CysPAT. The CysPAT strategy has several advantageous features such as (1) it is easy and cheap to synthesize the tagging reagent, (2) we observed an almost complete labeling of the Cys peptides with minor detectable side reactions, (3) high enrichment efficiency using TiO_2_, (4) no side effects on peptide solubility, chromatographic and MS/MS behavior, and (5) the possibility of simultaneous characterization of different PTMs which has an affinity toward TiO_2_, such as phosphopeptides and sialylated glycopeptides, offer the possibility to characterize the cross-talk of these PTMs from the same sample.

In the study of the brief EGF stimulation of HeLa cells presented here we clearly show a substantial modulation of reversible modified Cys sites presumable caused by the increase in H_2_O_2_ production after EGFR stimulation. Very surprisingly, we observe more regulated Cys sites compared with phosphorylation sites in this well-studied signaling pathway and many of these are on protein associated with chromatin remodeling and DNA replication. Furthermore, we observe regulation of Cys sites in a large number of enzymes that are associated with dynamic PTMs supporting the idea of reversible Cys modification having substantial cross-talks with other PTMs. Most of the regulated Cys sites have never been associated with EGF signaling before and this could open up for various new ideas to manipulate this pathway, which is very central in diseases such as cancer.

Overall, we believe that the concept of “phospho-based adaptable tags” approach will be a valuable tool for researchers and will be explored and extended to label other PTMs and improve the current proteomic toolbox.

## Supplementary Material

Supplemental Data

## Footnotes

^1^ The abbreviations used are:

oxPTM: oxidative post-translational modification
Cys: Cysteine
CysPAT: Cysteine-specific Phosphonate Adaptable Tag
TiO_2_: titanium dioxide
SIA: N-Succinimidyl iodoacetate
2-AEP: 2-aminoethylphosphonic acid
TEAB: triethylammonium bicarbonate
TCEP: tris-(2-Carboxyethyl)phosphine
ICAT: isotope coded affinity tags
BST: biotin switch tag
TMT: tandem mass tags
LC: Liquid chromatography
MS: mass spectrometry
HILIC: hydrophilic interaction liquid chromatography
BSA: bovine serum albumin
GAPDH: glyceraldehyde-3-phosphate dehydrogenase
PTPs: Protein-tyrosine phosphatases.
