# Supplementary material for: Simultaneous Enrichment of Cysteine-containing Peptides and Phosphopeptides Using a Cysteine-specific Phosphonate Adaptable Tag (CysPAT) in Combination with titanium dioxide (TiO2) Chromatography
Source: Mol Cell Proteomics. 2016 Oct;15(10):3282–96. doi: 10.1074/mcp.M115.054551 (PMC5054350; doi:10.1074/mcp.M115.054551)
Supplement: Supplemental Data [file supp_15_10_3282__index.html]

Simultaneous enrichment of cysteine-containing peptides and phosphopeptides using a Cysteine-specific Phosphonate Adaptable Tag (CysPAT) in combination with TiO2 chromatography — Simultaneous Enrichment of Cysteine-containing Peptides and Phosphopeptides Using a Cysteine-specific Phosphonate Adaptable Tag (CysPAT) in Combination with titanium dioxide (TiO2) Chromatography — Coenrichment of Cysteine-containing Peptides and Phosphopeptides With CysPAT and TiO2 — Supplemental Data 

# Simultaneous Enrichment of Cysteine-containing Peptides and Phosphopeptides Using a Cysteine-specific Phosphonate Adaptable Tag (CysPAT) in Combination with titanium dioxide (TiO2) Chromatography

## Supplemental Data

- Figures (.pdf, 1.8 MB) - Figures
- Supplementary Figures legend (.pdf, 93 KB) - Supplementary Figures legend
- Supplementary Figures (.pdf, 2.1 MB) - Supplementary Figures
- Supplementary Tables (.xlsx, 10.5 MB) - Supplementary Tables
