# Supplementary material for: Simultaneous Enrichment of Cysteine-containing Peptides and Phosphopeptides Using a Cysteine-specific Phosphonate Adaptable Tag (CysPAT) in Combination with titanium dioxide (TiO2) Chromatography
Source: Mol Cell Proteomics. 2016 Oct;15(10):3282–96. doi: 10.1074/mcp.M115.054551 (PMC5054350; doi:10.1074/mcp.M115.054551)
Supplement: Supplemental Data [file 10.1074_M115.054551_mcp.M115.054551-1.pdf]

Figure 1

a)

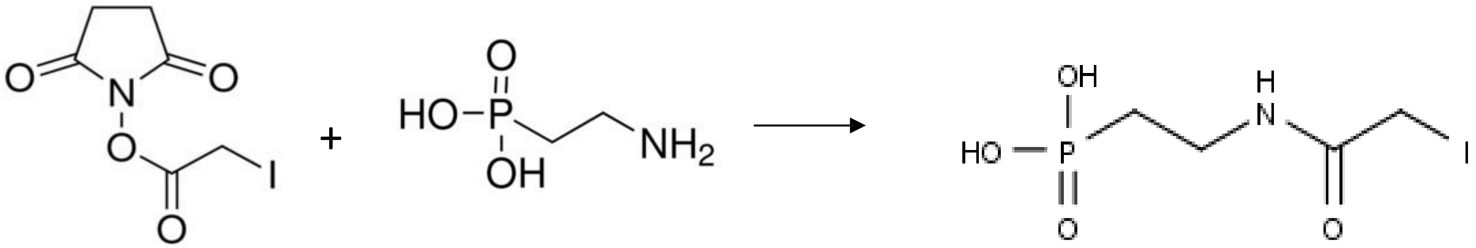

Succinimidyl iodoacetate(SIA) MW: 283,02

2-aminoethyl)phosphonic acid (AEP) MW: 125,06

(2-(2-iodoacetamido)ethyl)phosphonic acid MW: 292,93

b)

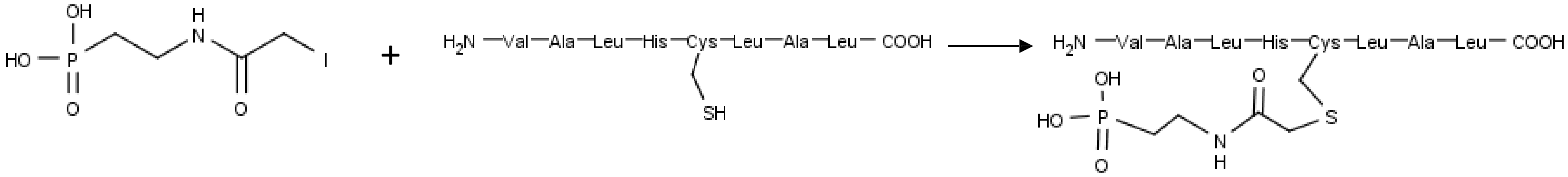

Figure 2

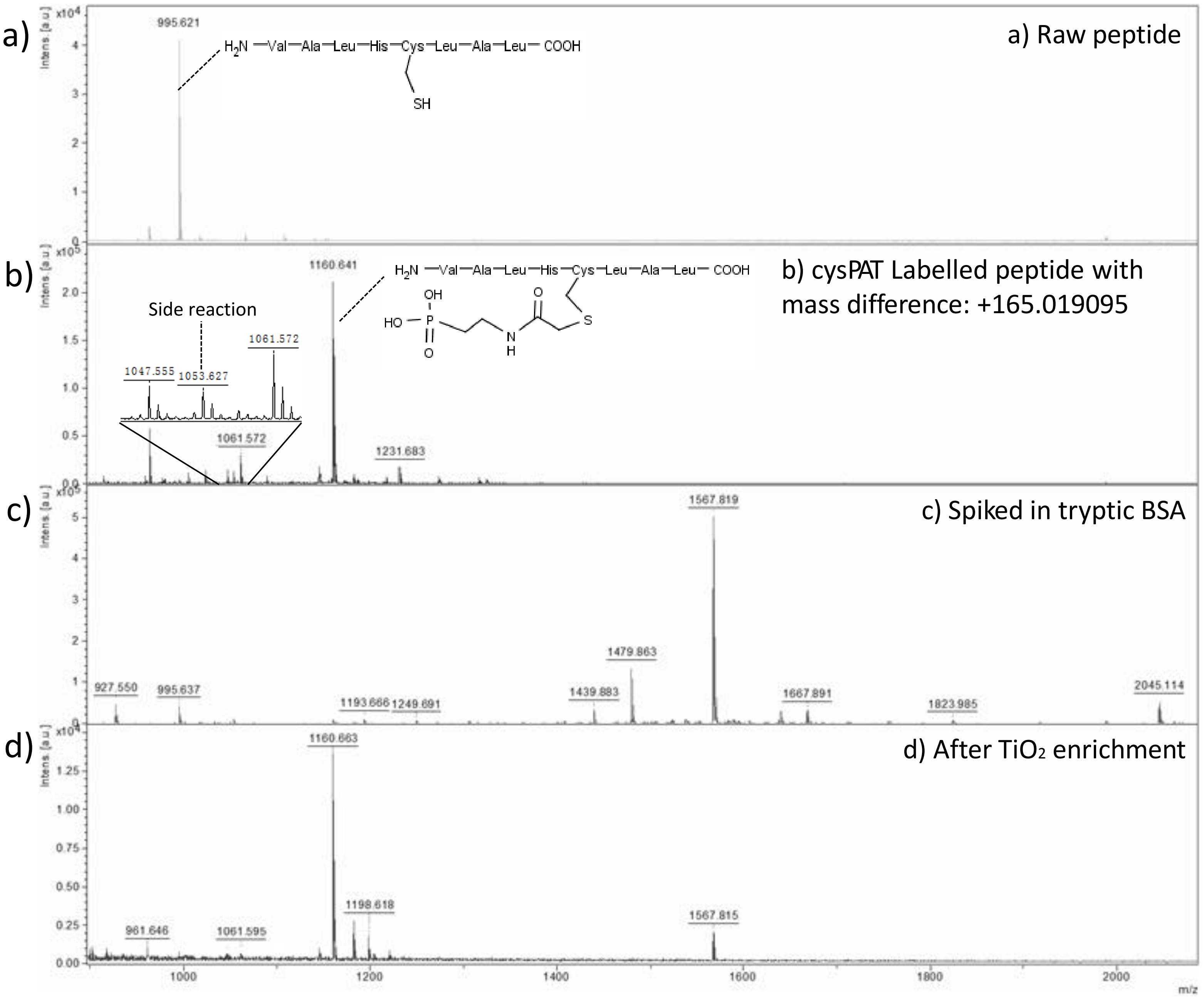

a)
a) Raw peptide
995.621
H2N —Val—Ala—Leu—His—Cys—Leu—Ala—Leu— COOH
SH
b)
b) cysPAT Labelled peptide with
mass difference: +165.019095
1160.641
Side reaction
1047.555
1053.627
1061.572
1061.572
1231.683
H2N —Val—Ala—Leu—His—Cys—Leu—Ala—Leu— COOH
OH
HO — P
O
N
H
S
c)
c) Spiked in tryptic BSA
1567.819
1479.863
927.550
995.637
1193.666
1249.691
1439.883
1667.891
1823.985
2045.114
d)
d) After TiO2 enrichment
1160.663
1198.618
961.646
1061.595
1567.815
Intens. [a.u.]
m/z

Figure 3

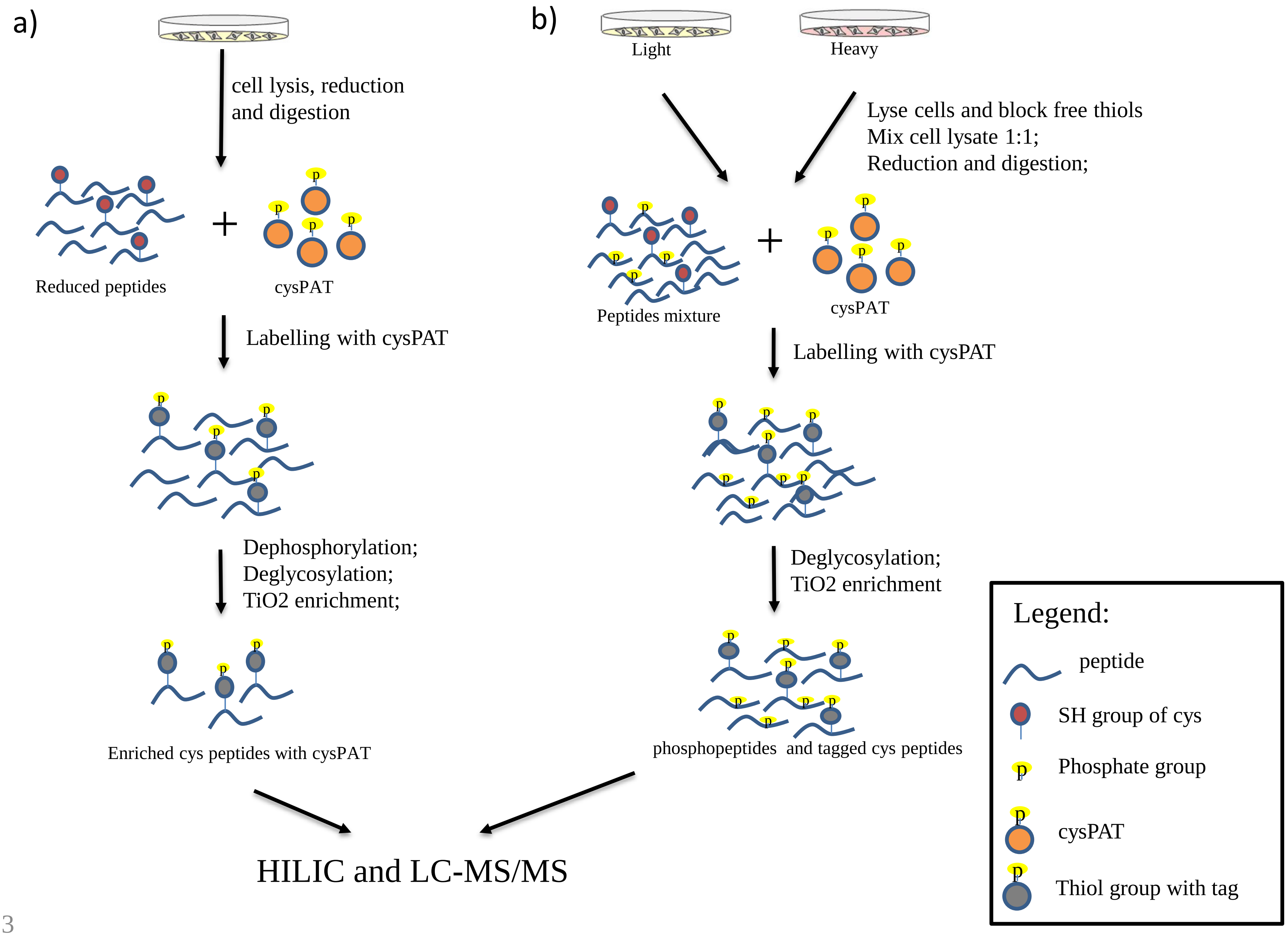
a)
cell lysis, reduction
and digestion
Reduced peptides
cysPAT
Labelling with cysPAT
Dephosphorylation;
Deglycosylation;
TiO2 enrichment;
Enriched cys peptides with cysPAT
b)
control
5 min EGF
Light
Heavy
Lyse cells and block free thiols
Mix cell lysate 1:1;
Reduction and digestion;
Peptides mixture
cysPAT
Labelling with cysPAT
Deglycosylation;
TiO2 enrichment
phosphopeptides and tagged cys peptides
HILIC and LC-MS/MS
Legend:
peptide
SH group of cys
Phosphate group
cysPAT
Thiol group with tag

Figure 4

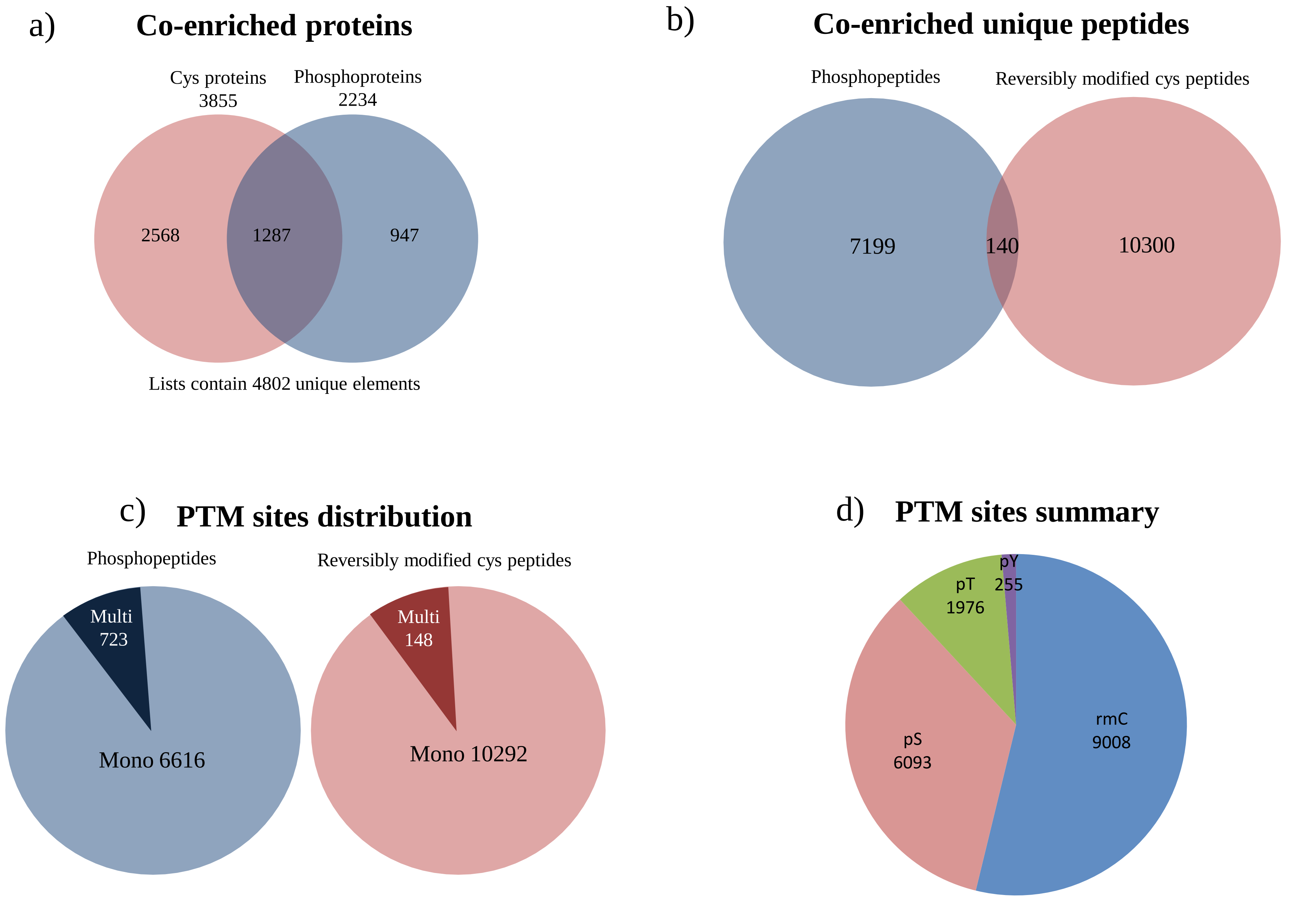
a)
Co-enriched proteins
Cys proteins
3855
Phosphoproteins
2234
2568
1287
947
Lists contain 4802 unique elements
b)
Co-enriched unique peptides
Phosphopeptides
Reversibly modified cys peptides
7199
140
10300
c)
PTM sites distribution
Phosphopeptides
Multi
723
Mono 6616
Reversibly modified cys peptides
Multi
148
Mono 10292
d)
PTM sites summary
pY
255
pT
1976
pS
6093
rmC
9008

Figure 5

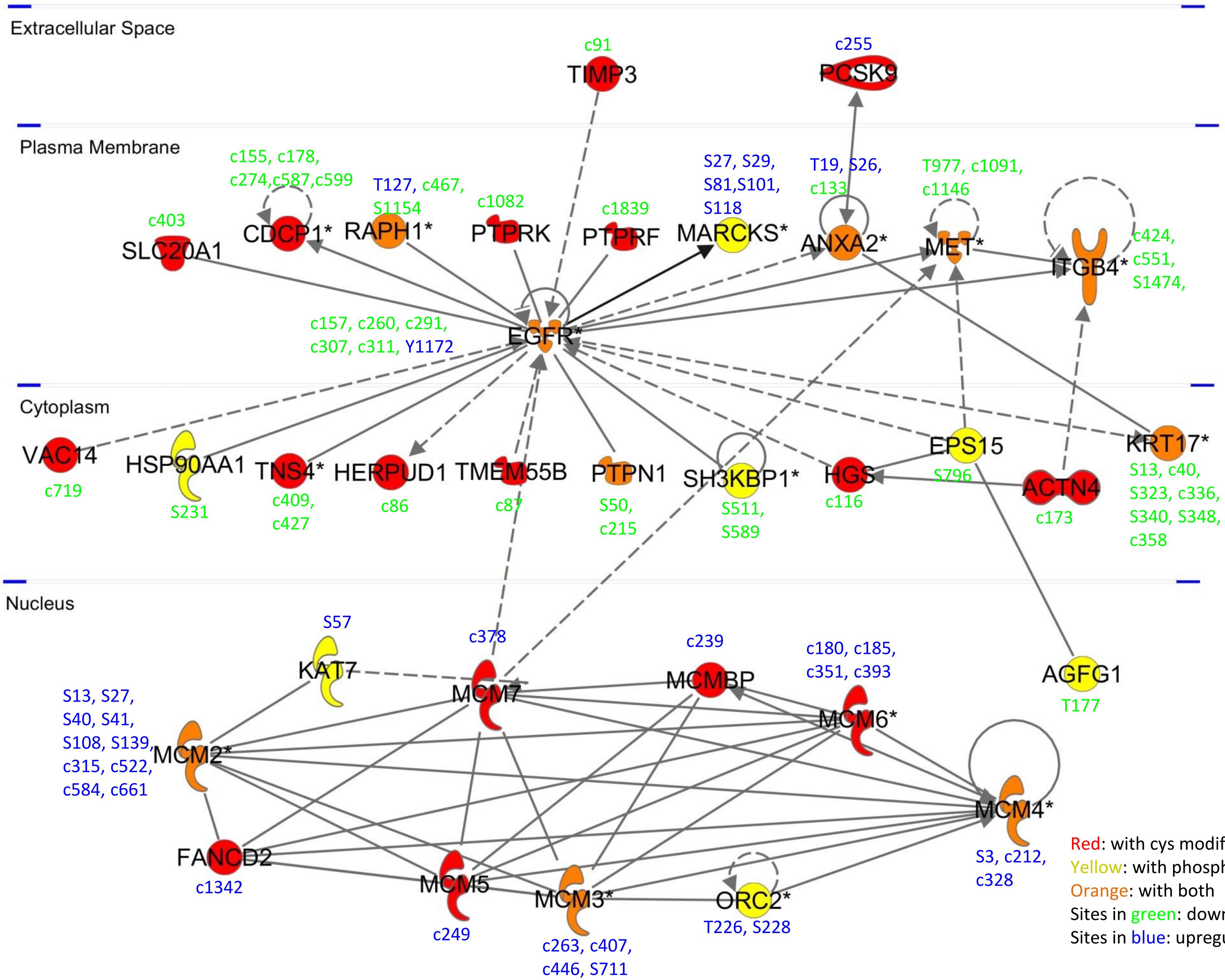

Red: with cys modification
Yellow: with phosphorylation
Orange: with both
Sites in green: downregulated
Sites in blue: upregulated

Figure 6

a)

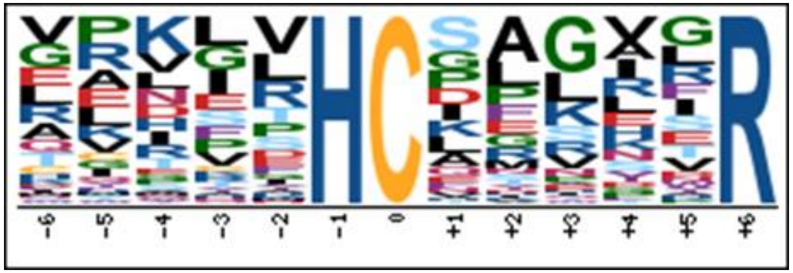

Peptides	PTPs	Location	log2(H/L)	STD
**ESGSLSPEHGPVVVH*CSAGIGR**	**PTPN1**	**C215**	**-1.08**	**0.32**
**EQFGQDGPITVH*CSAGVGR**	**PTPRF**	**C1839**	**-2.37**	**0.32**
**LSNPPSAGPIVVH*CSAGAGR**	**PTPRK**	**C1082**	**-2.30**	**0.24**
GEAVGVH*CALGFGR	DUSP23	C95	-1.97	NA
ESGSLNPDHGPAVIH*CSAGIGR	PTPN2	C216	0.21	NA
VKACNPQYAGAIVVH*CSAGVGR	PTPRA	C442	-0.05	NA
TIIH*CLNGGGR	PTPRK	C1376	NA	NA

b)

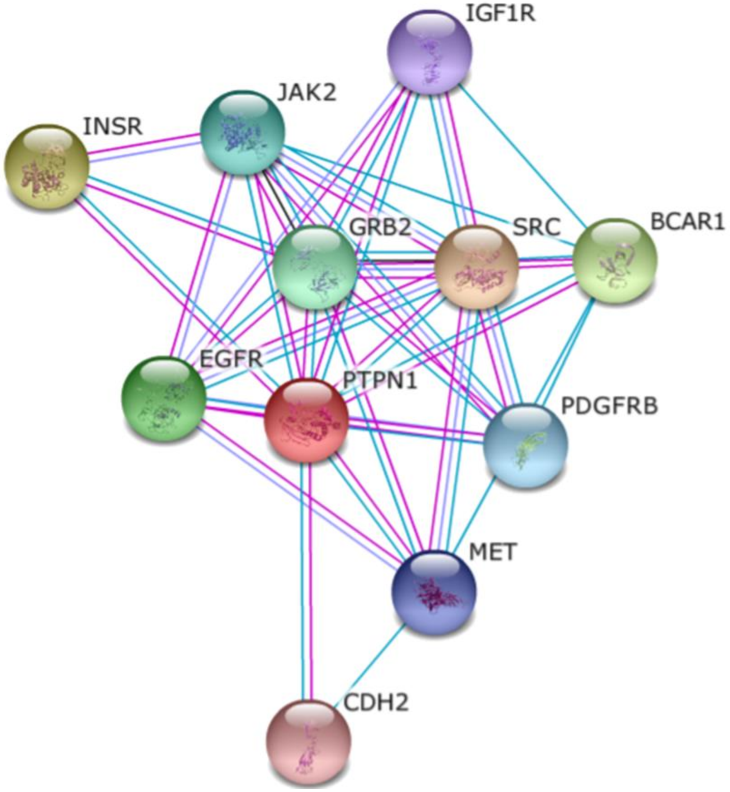

c)

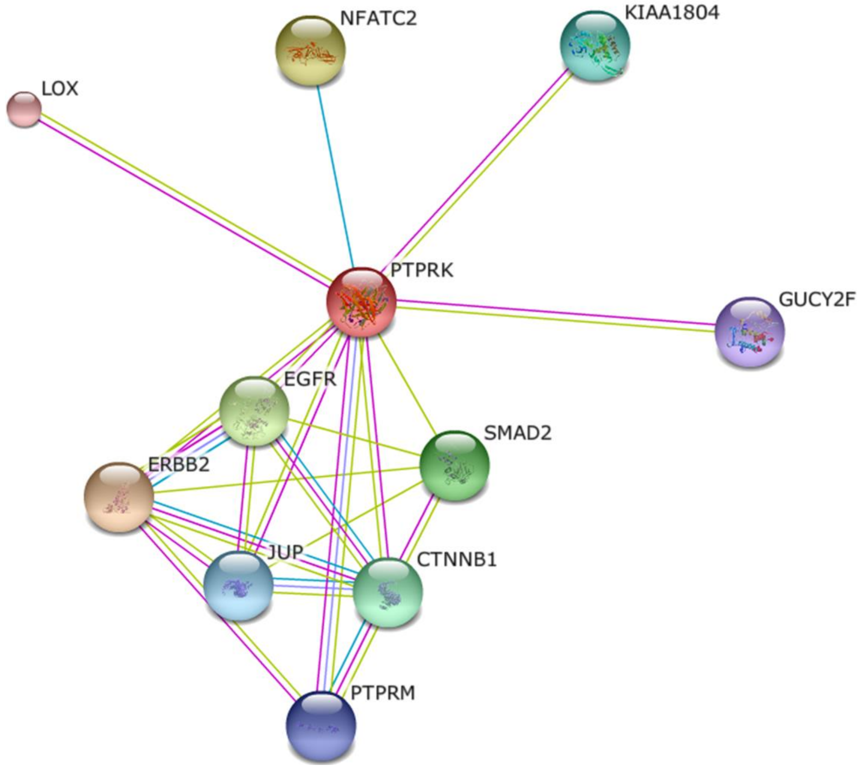

Figure 7

Up-regulated SIA proteins

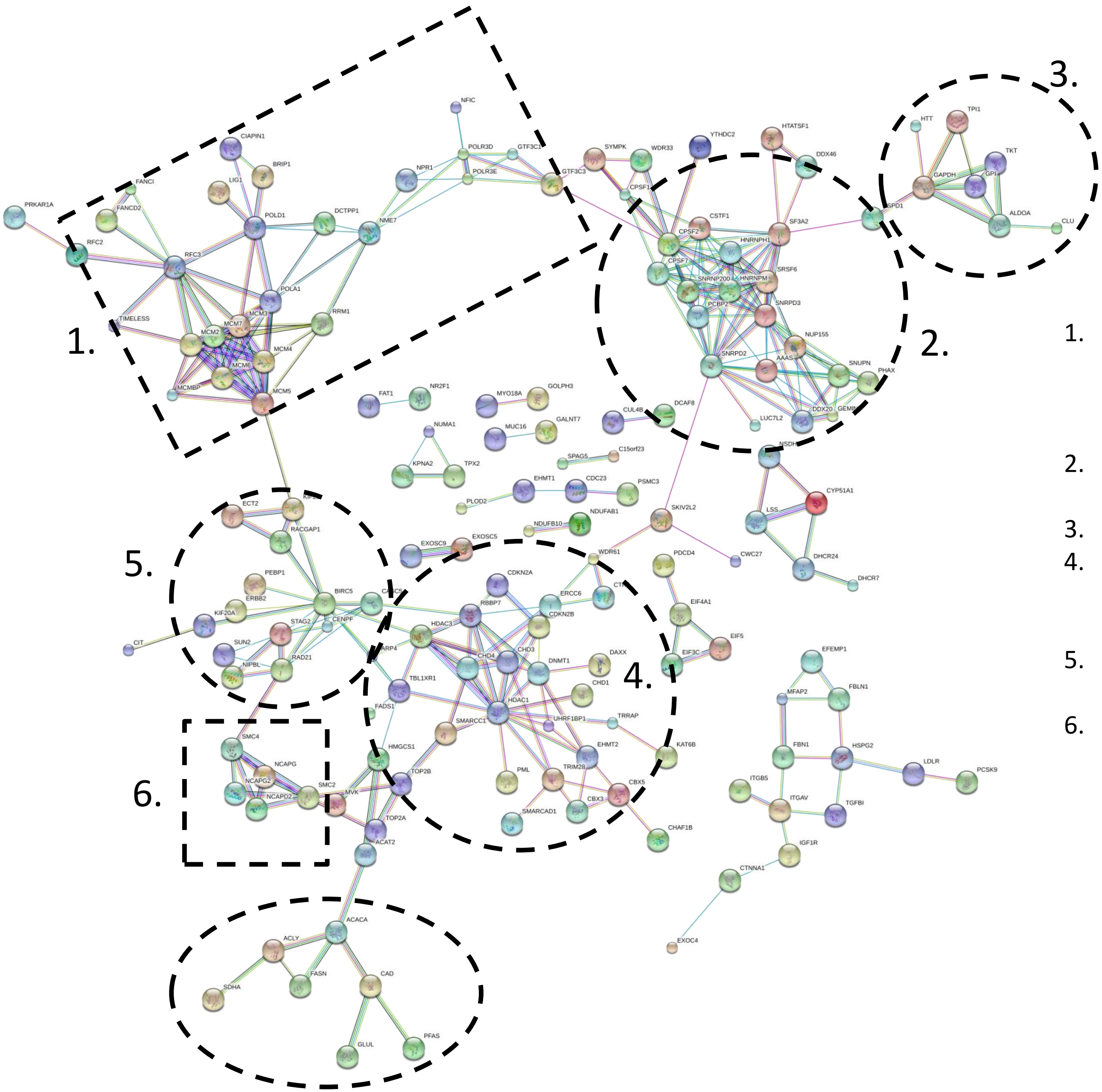

1. DNA replication, incl transctiption factors, polymerases and the Minichromosome maintenance complex
2. Pre-mRNA processing transportation and splicing
3. Glycolysis
4. Chromatin remodeling, incl deacetylases and DNA methyltransferases
5. Cytokinesis and sister chromatid cohesion.
6. DNA condensin complex

Figure 8

Down-regulated SIA proteins

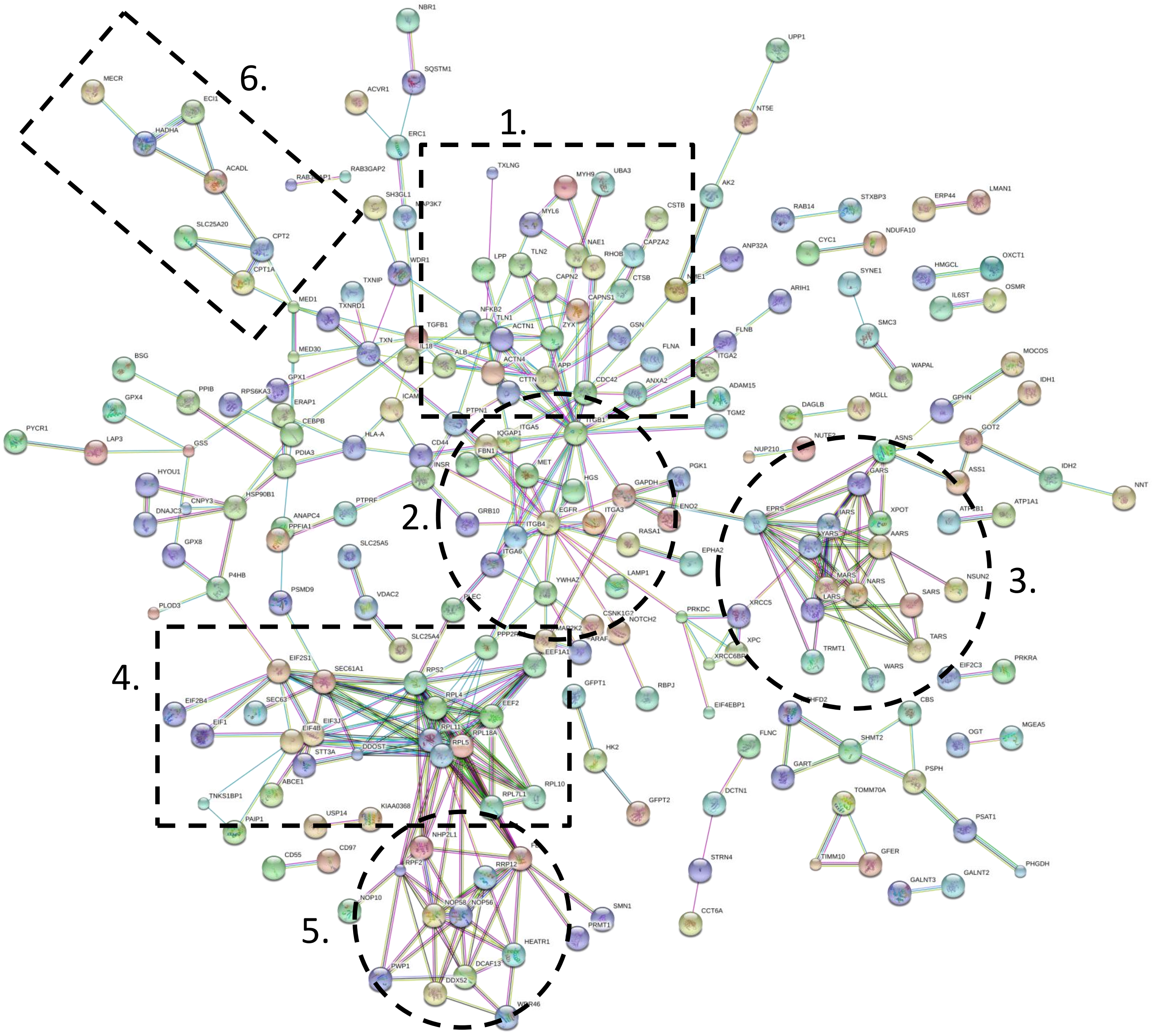

1. Integrin signaling with the cytoskeleton networks
2. EGFR signaling molecules
3. tRNA synthetases
4. Protein translation; ribosomes, initiation and elongation factors
5. Ribosomal biosynthesis and RNA splicing
6. fatty acid-oxidation
