# Supplementary material for: Simultaneous Enrichment of Cysteine-containing Peptides and Phosphopeptides Using a Cysteine-specific Phosphonate Adaptable Tag (CysPAT) in Combination with titanium dioxide (TiO2) Chromatography
Source: Mol Cell Proteomics. 2016 Oct;15(10):3282–96. doi: 10.1074/mcp.M115.054551 (PMC5054350; doi:10.1074/mcp.M115.054551)
Supplement: Supplemental Data [file 10.1074_M115.054551_mcp.M115.054551-2.pdf]

Simultaneous enrichment of cysteine-containing peptides and phosphopeptides using a Cysteine-specific Phosphonate Adaptable Tag (CysPAT) in combination with TiO_2 chromatography

Honggang Huang, Martin Haar Petersen, Maria Ibanez Vea, Pernille S. Lassen, Martin R. Larsen and Giuseppe Palmisano

Supplementary figures and tables.

Supplementary Figure S1: a) MALDI-MS/MS spectrum of CysPAT-labeled standard peptide (VALRHCLAL). b) ESI-MS/MS spectrum of the BSA-derived peptide labeled with CysPAT. The presence of a diagnostic ion at 224.01 m/z is shown. c) Possible structure of the CysPAT diagnostic ion.

Supplementary Figure S2: All the Cys sites in BSA were identified after enrichment from CysPAT labeled BSA peptides.

Supplementary Figure S3: a) Charge state distribution and b) hydrophobicity of peptides after CysPAT or IAA labelling.

Supplementary Figure S4: The differential elution pattern of phosphopeptides and CysPAT labelled Cys peptides in HILIC chromography. The number of peptides reported include the other variable modifications used for database search.

Supplementary Figure S5: The statistical summary of regulated peptides and proteins.

Supplementary Figure S6: MS and MS/MS spectra of the CysPAT-tagged peptide (GVV*CIDEFDK) belonging to DNA replication licensing factor MCM3 protein. *C indicates the CysPAT-tagged cysteine. CysPAT* at 224.01 m/z indicates the CysPAT diagnostic ion.

Supplementary Figure S7: MS and MS/MS spectra of the CysPAT-tagged peptide (ESGSLSPEHGPVVVH*CSAGIGR) belonging to Tyrosine-protein phosphatase non-receptor type 1 (PTP1B/PTPN1) protein. *C indicates the CysPAT-tagged cysteine. CysPAT* at 224.01 m/z indicates the CysPAT diagnostic ion.

Supplementary Figure S8: MS and MS/MS spectra of the phosphorylated and CysPAT-tagged peptide (RAP*SVANVGSH*CDLSLK) belonging to Filamin A protein. *S indicates the phosphorylated site and *C indicated the CysPAT-tagged cysteine. CysPAT* at 224.01 m/z indicates the CysPAT diagnostic ion.

Supplementary Figure S9: Protein-protein interaction network of protein tyrosine phosphatase type IVA 1 using STRING.

Supplementary Figure S10: The proteins identified with PTM sites in the EGFR signaling pathway. Proteins identified with PTM sies were marked in color, in red: with only Cys modification; in yellow: with only phosphorylation; in orange: with both PTMs. Only the significantly regulated PTM sites were indicated in color, in green: downregulated; in blue: upregulated.

Supplementary Figure S11: Protein-protein interaction network of mitochondrial proteins identified with regulated reversible Cys residues using STRING.

Supplementary Figure S12: Protein-protein interaction network of redox related proteins with regulated reversible Cys residues using STRING.

Supplementary Figure S13: Protein-protein interaction network of ubiquitinylation and neddylation related proteins identified with regulated reversible Cys

Supplementary Table S1: List of identified unique peptides from Hela cell lysate enriched for the total cysteine experiment. Peptides sequences with CysPAT labeling, indicated as SIA, and N-terminal protein acetylation are reported. Database search identification output reports protein groups, PSMs, number of CysPAT labeling, missed cleavage, m/z, retention time, q-value, Ion score, rank and search engine rank.

Supplementary Table S2: List of identified unique peptides from Hela cell lysate enriched for the reversibly modified cysteines. Peptides sequences with CysPAT labeling, indicated as SIA, and N-terminal protein acetylation are reported. Database search identification output reports protein groups, PSMs, number of CysPAT labeling, missed cleavage, m/z, retention time, q-value, Ion score, rank and search engine rank.

Supplementary Table S3: List of identified unique peptides by the simultaneous enrichment of reversibly modified Cys peptides and phosphopeptides from SILAC labelled Hela cells subject to 5 min EGF stimulation. Peptides sequences with CysPAT labeling, indicated as SIA, and N-terminal protein acetylation are reported. Database search identification output reports protein groups, PSMs, number of CysPAT labeling, missed cleavage, m/z, retention time, q-value, Ion score, rank, search engine rank, phosphoRS score.

Supplementary Table S4: List of mapped reversibly modified Cys sites and phosphorylation sites with information of localization on protein, motif window and site annotation retrieved from Uniprot database

Supplementary Table S5: List of significantly regulated peptides with PTM sites and statistical information.

Supplementary Table S6: List of significantly regulated peptides from proteins in the EGFR network and EGFR signaling pathway.

Supplementary Table S7: List of identified peptides with HC(X5)R motif and related sites and protein information.
