# Supplementary material for: Simultaneous Enrichment of Cysteine-containing Peptides and Phosphopeptides Using a Cysteine-specific Phosphonate Adaptable Tag (CysPAT) in Combination with titanium dioxide (TiO2) Chromatography
Source: Mol Cell Proteomics. 2016 Oct;15(10):3282–96. doi: 10.1074/mcp.M115.054551 (PMC5054350; doi:10.1074/mcp.M115.054551)

Supplementary Figure S1

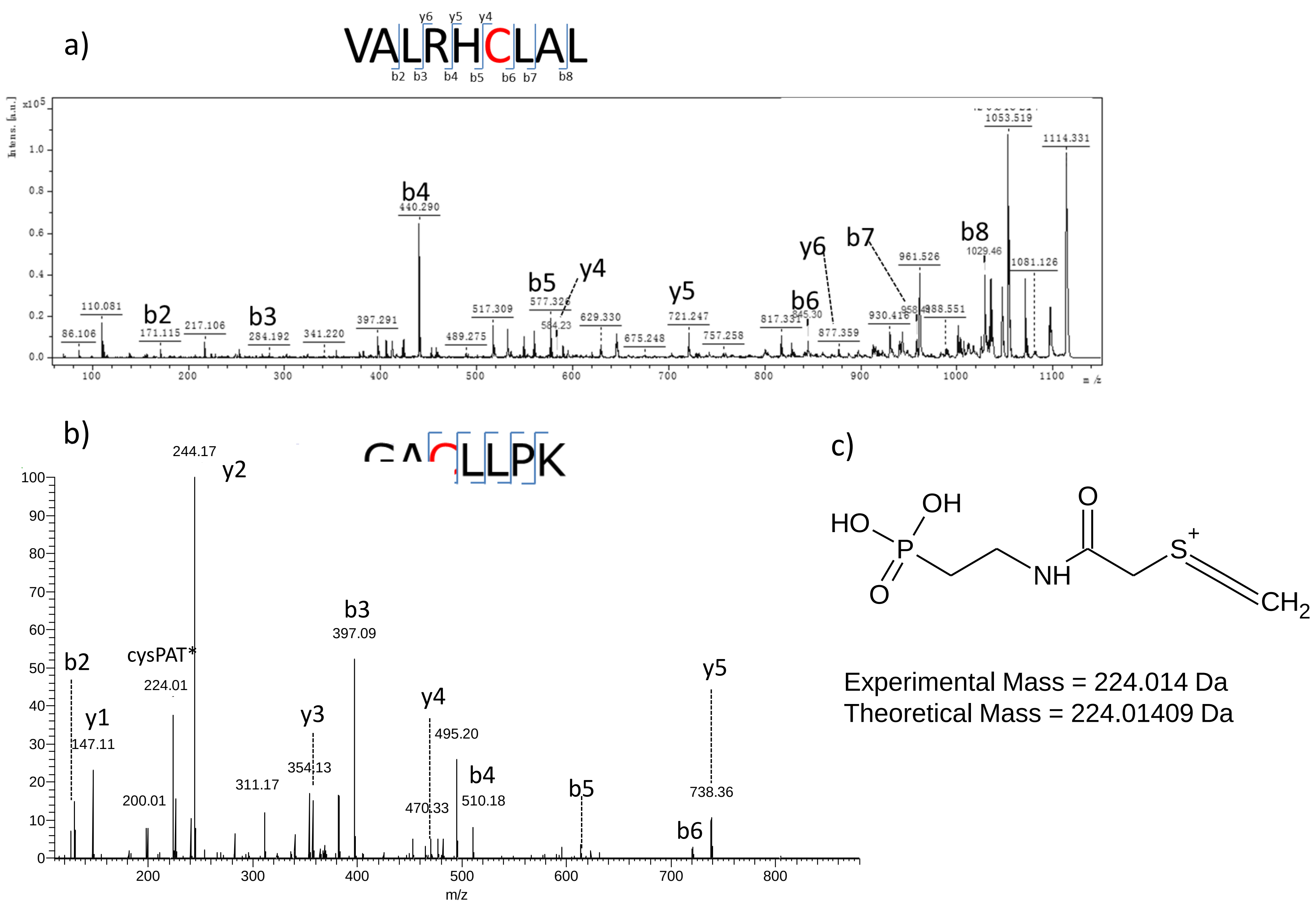

Supplementary Figure S2

P02769 (ALBU_BOVIN) Serum albumin

MKWVTFISLLLLFSSAYSRGVFRRDTHKSEIAHRFKDLGEEHFKGLVLIAFSQYLQQcPFDEHVKLV

NELTEFAKTcVADESHAGcEKSLHTLFGDELcKVASLRETYGDMADccEKQEPERNEcFLSHKDDS

PDLPKLKPDPNTLcDEFKADEKKFWGKYLYEIARRHPYFYAPELLYYANKYNGVFQEccQAEDKGA

cLLPKIETMREKVLASSARQRLRcASIQKFGERALKAWSVARLSQKFPKAEFVEVTKLVTDLTKVHK

EccHGDLLEcADDRADLAKYIcDNQDTISSKLKEccDKPLLEKSHcIAEVEKDAIPENLPPLTADFAED

KDVcKNYQEAKDAFLGSFLYEYSRRHPEYAVSVLLRLAKEYEATLEEccAKDDPHAcYSTVFDKLKH

LVDEPQNLIKQNcDQFEKLGEYGFQNALIVRYTRKVPQVSTPTLVEVSRSLGKVGTRccTKPESER

MPcTEDYLSLILNRLcVLHEKTPVSEKVTKccTESLVNRRPcFSALTPDETYVPKAFDEKLFTFHADIc

TLPDTEKQIKKQTALVELLKHKPKATEEQLKTVMENFVAFVDKccAADDKEAcFAVEGPKLVVSTQTA

LA

Supplementary Figure S3

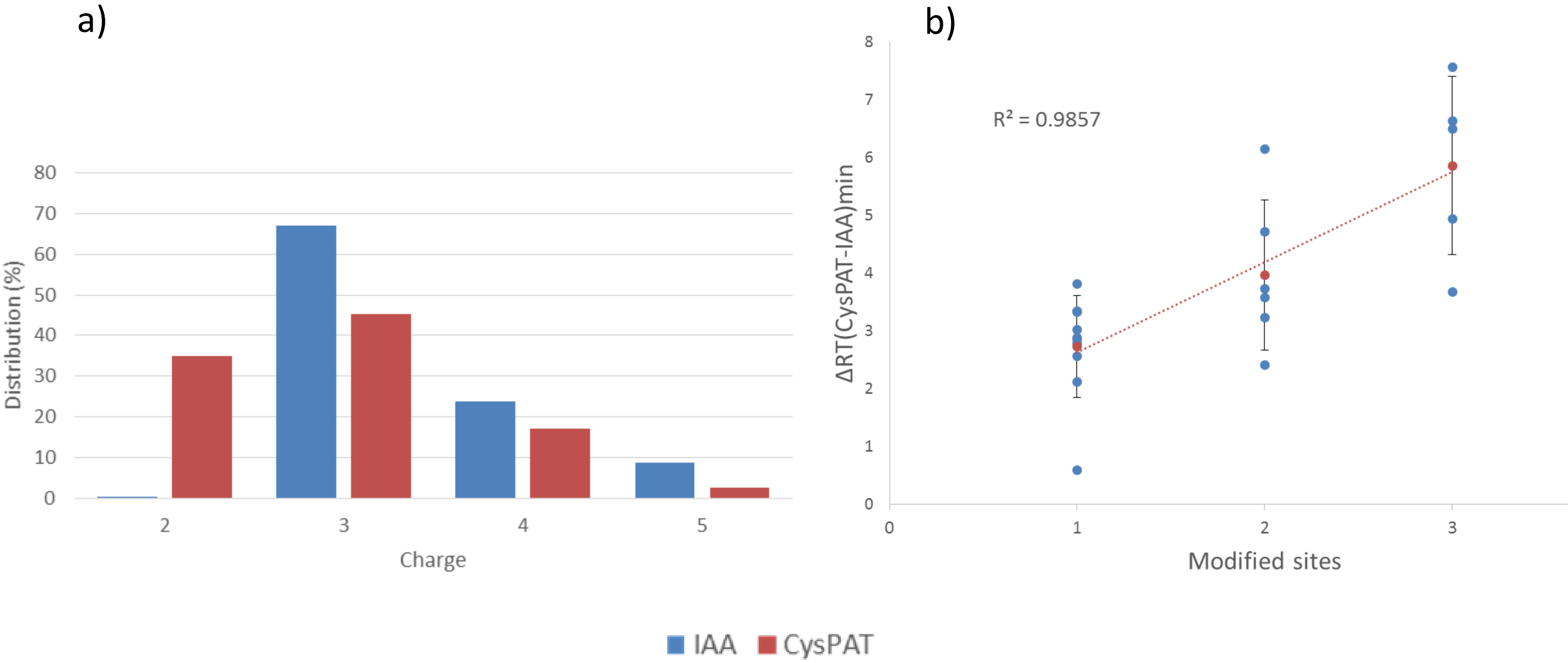

Supplementary Figure S4

HILIC fractionation

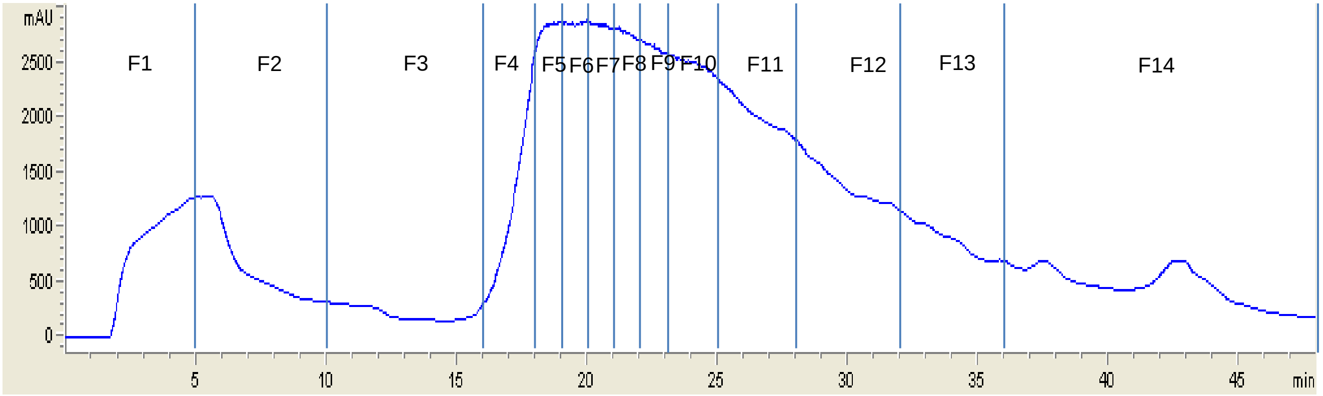

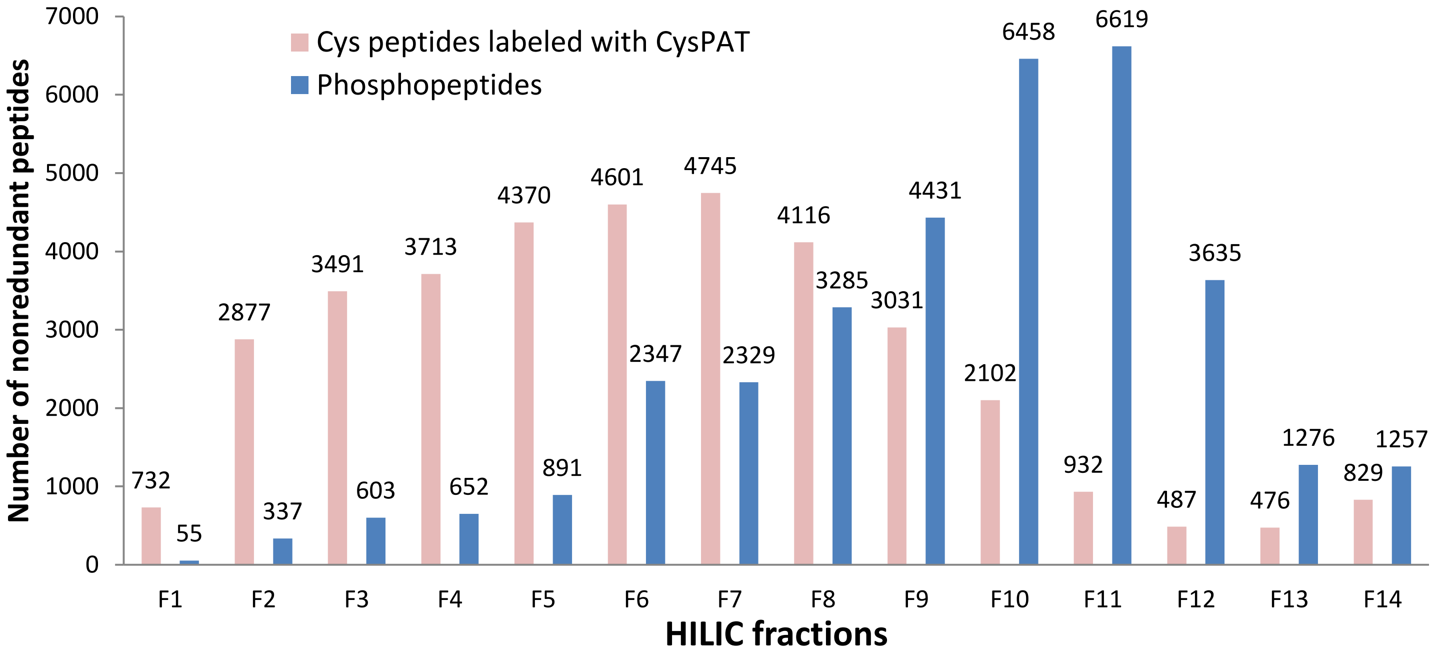

Supplementary Figure S5

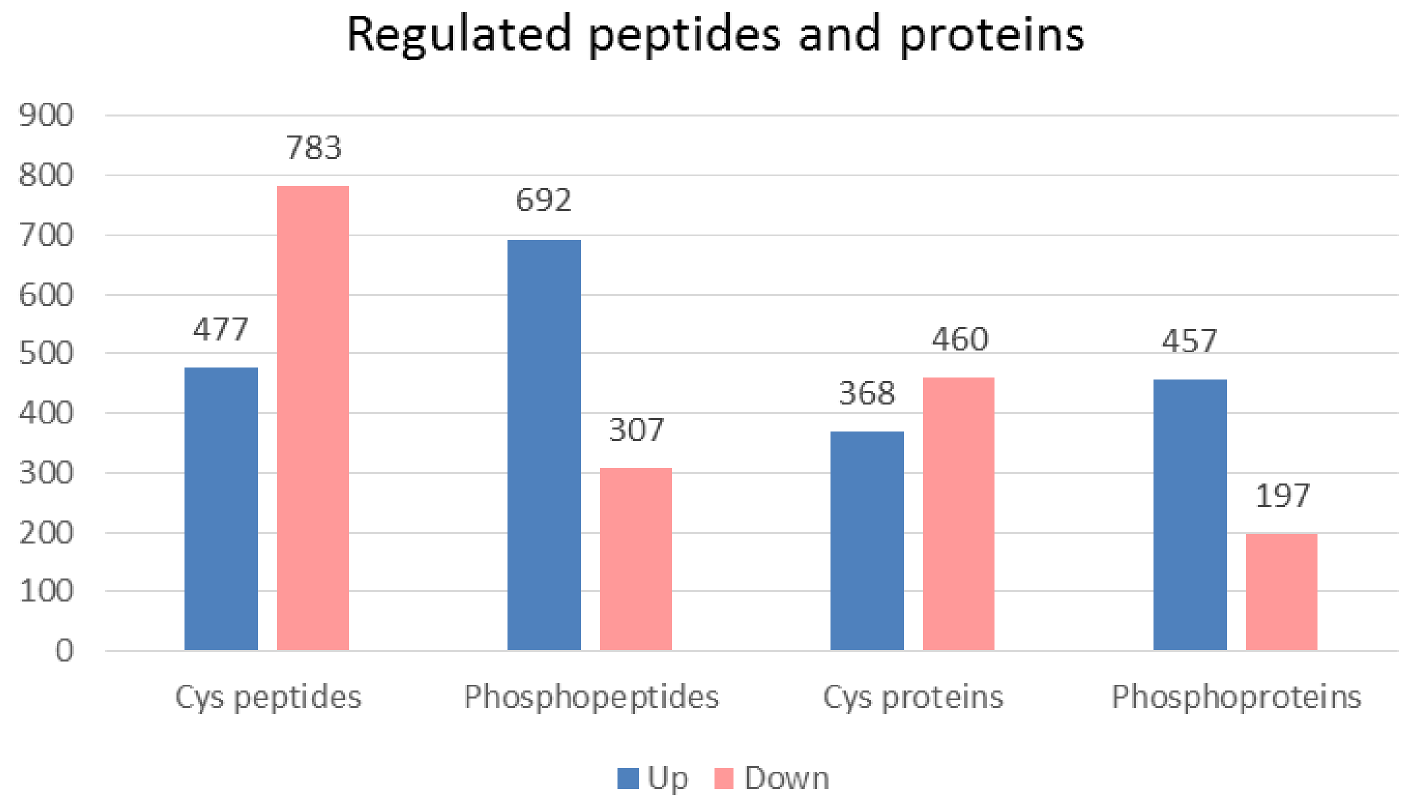

Supplementary Figure S6

Cys407 of MCM3
GVVCIDEFDK,
m/z=645.28, z=2

Sequence	Protein	Site	Mean of log2 (H/L)	STD of log
GVV*CIDEFDK	MCM3	C407	0.98	0.11

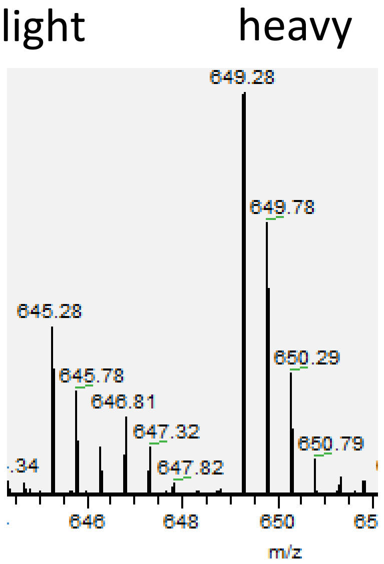

#1	b⁺	b²⁺	Seq.	y⁺	y²⁺	#2
1	58.02875	29.51801	G			10
2	157.09717	79.05222	V	1232.52693	616.76710	9
3	256.16559	128.58643	V	1133.45851	567.23289	8
4	524.19387	262.60057	C-SIA	1034.39009	517.69868	7
5	637.27794	319.14261	I	766.36180	383.68454	6
6	752.30489	376.65608	D	653.27773	327.14250	5
7	881.34749	441.17738	E	538.25078	269.62903	4
8	1028.41591	514.71159	F	409.20818	205.10773	3
9	1143.44286	572.22507	D	262.13976	131.57352	2
10			K	147.11281	74.06004	1

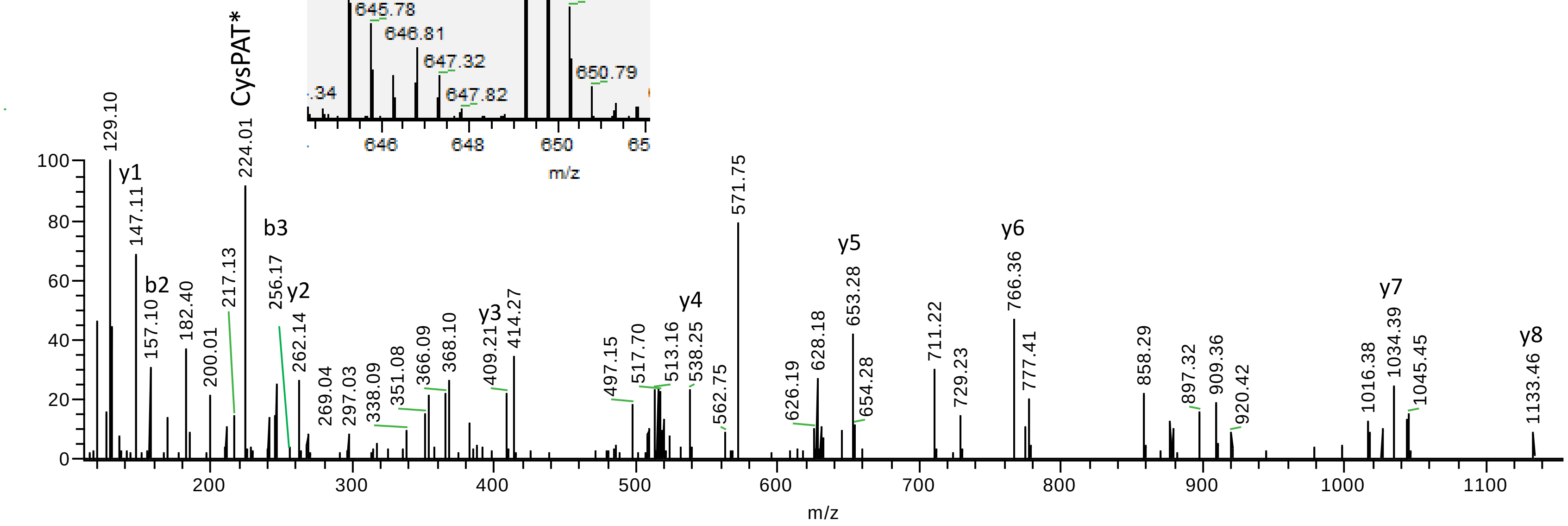

Supplementary Figure S7

Cys215 of PTP1B/PTPN1 ESGSLSPEHGPVVVHCSAGIGR, m/z=780.70, z=3

Peptides	PTPs	Location	log2(H/L)	STD
ESGSLSPEHGPVVVH*CSAGIGR	PTPN1	C215	-1.08	0.32

#1	b⁺	b²⁺	b³⁺	Seq.	y⁺	y²⁺	y³⁺	#2
1	130.04988	65.52858	44.02148	E				22
2	217.08191	109.04459	73.03215	S	2211.03773	1106.02250	737.68409	21
3	274.10338	137.55533	92.03931	G	2124.00570	1062.50649	708.67342	20
4	361.13541	181.07134	121.04999	S	2066.98423	1033.99575	689.66626	19
5	474.21948	237.61338	158.74468	L	1979.95220	990.47974	660.65558	18
6	561.25151	281.12939	187.75535	S	1866.86813	933.93770	622.96089	17
7	658.30428	329.65578	220.10628	P	1779.83610	890.42169	593.95022	16
8	787.34688	394.17708	263.12048	E	1682.78333	841.89530	561.59929	15
9	924.40579	462.70653	308.80678	H	1553.74073	777.37400	518.58509	14
10	981.42726	491.21727	327.81394	G	1416.68182	708.84455	472.89879	13
11	1078.48003	539.74365	360.16486	P	1359.66035	680.33381	453.89163	12
12	1177.54845	589.27786	393.18767	V	1262.60758	631.80743	421.54071	11
13	1276.61687	638.81207	426.21047	V	1163.53916	582.27322	388.51790	10
14	1375.68529	688.34628	459.23328	V	1064.47074	532.73901	355.49510	9
15	1512.74420	756.87574	504.91958	H	965.40232	483.20480	322.47229	8
16	1780.77248	890.88988	594.26234	C-SIA	828.34341	414.67534	276.78599	7
17	1867.80451	934.40589	623.27302	S	560.31512	280.66120	187.44322	6
18	1938.84163	969.92445	646.95206	A	473.28309	237.14518	158.43255	5
19	1995.86310	998.43519	665.95922	G	402.24597	201.62662	134.75351	4
20	2108.94717	1054.97722	703.65391	I	345.22450	173.11589	115.74635	3
21	2165.96864	1083.48796	722.66106	G	232.14043	116.57385	78.05166	2
22				R	175.11896	88.06312	59.04450	1

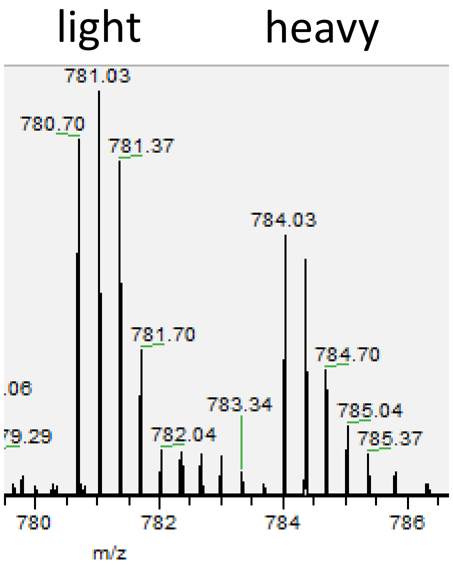

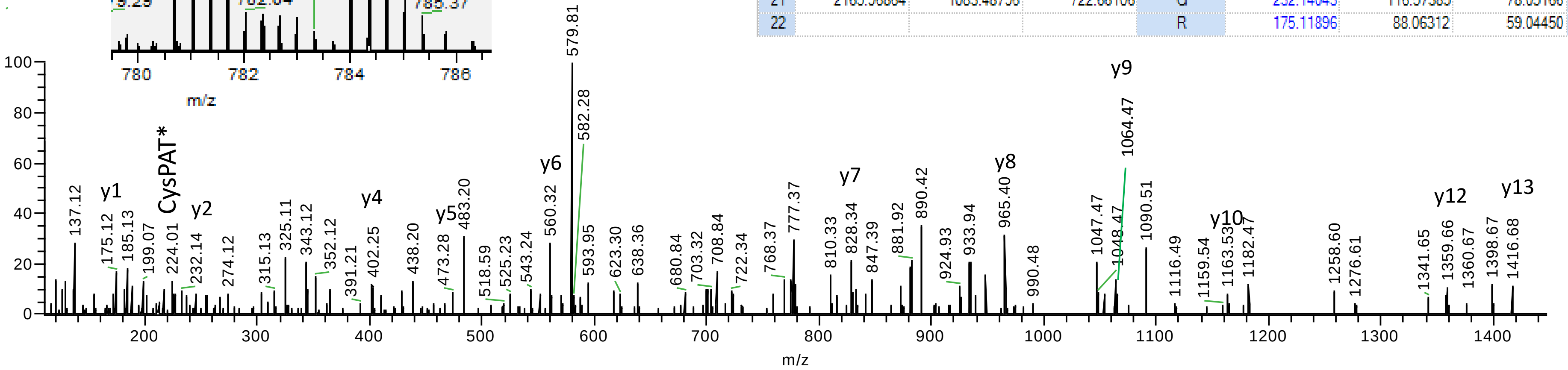

Supplementary Figure S8

S2152 and C2160 from Filamin A
RAPSVANVGSHCDLSLK
m/z=666.97, z=3

Sequence PTM	Sites	Protein Gr	Mean of log2 (H/L)	STD of log
RAP*SVANVGSH*CDLSLK	S2152, C2160	P21333	-1.23	0.15

#1	b⁺	b²⁺	b³⁺	Seq.	y⁺	y²⁺	y³⁺	#2
1	157.10840	79.05784	53.04098	R				17
2	228.14552	114.57640	76.72002	A	1842.78577	921.89652	614.93344	16
3	325.19829	163.10278	109.07095	P	1771.74865	886.37796	591.25440	15
4	492.19665	246.60196	164.73707	S-Phosp	1674.69588	837.85158	558.90348	14
5	591.26507	296.13617	197.75987	V	1507.69752	754.35240	503.23736	13
6	662.30219	331.65473	221.43891	A	1408.62910	704.81819	470.21455	12
7	776.34512	388.67620	259.45322	N	1337.59198	669.29963	446.53551	11
8	875.41354	438.21041	292.47603	V	1223.54905	612.27816	408.52120	10
9	932.43501	466.72114	311.48319	G	1124.48063	562.74395	375.49839	9
10	1019.46704	510.23716	340.49386	S	1067.45916	534.23322	356.49124	8
11	1156.52595	578.76661	386.18017	H	980.42713	490.71720	327.48056	7
12	1424.55423	712.78075	475.52293	C-SIA	843.36822	422.18775	281.79426	6
13	1539.58118	770.29423	513.86525	D	575.33993	288.17360	192.45149	5
14	1652.66525	826.83626	551.55994	L	460.31298	230.66013	154.10918	4
15	1739.69728	870.35228	580.57061	S	347.22891	174.11809	116.41449	3
16	1852.78135	926.89431	618.26530	L	260.19688	130.60208	87.40381	2
17				K	147.11281	74.06004	49.70912	1

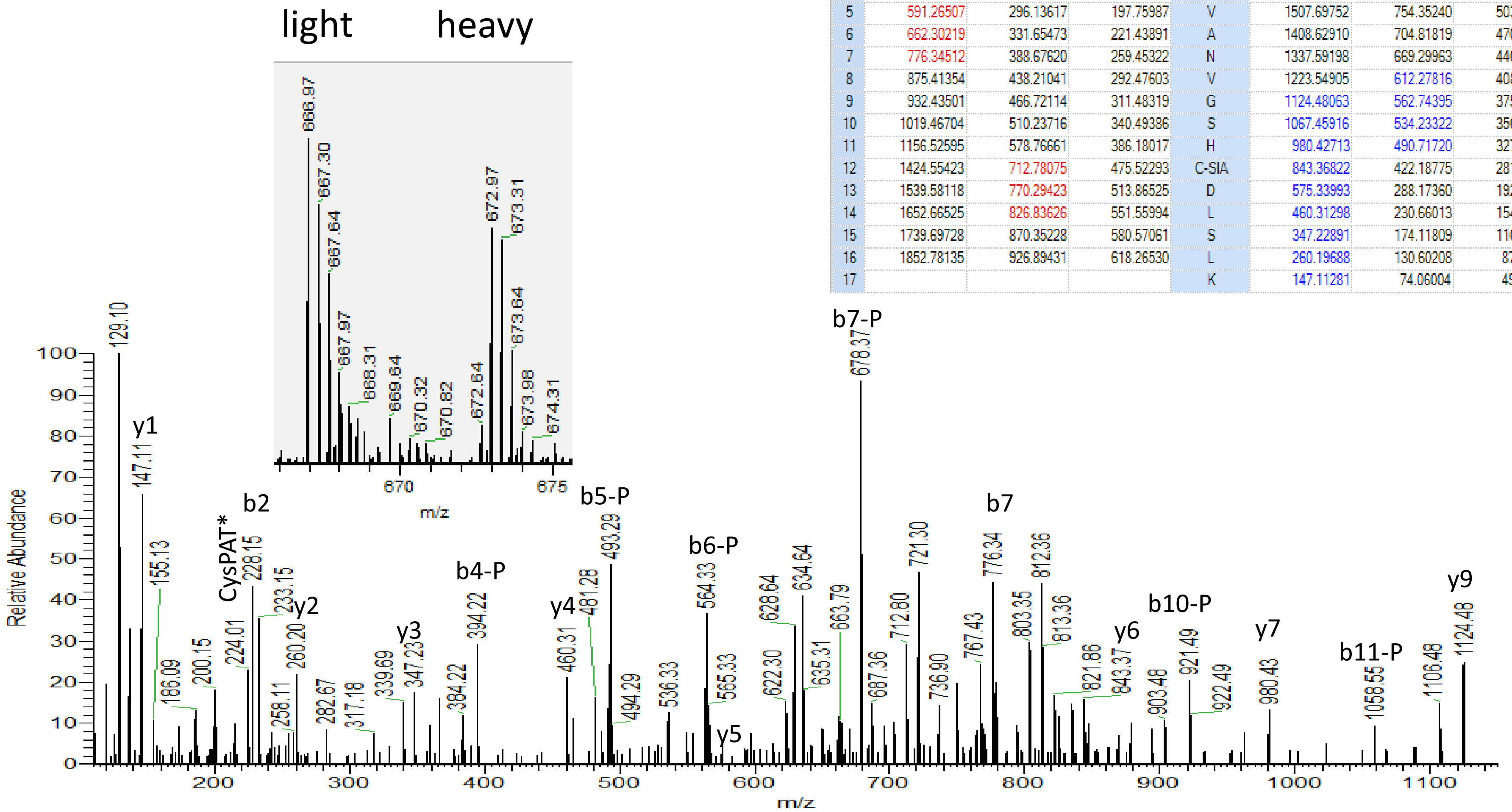

Supplementary Figure S9

Protein tyrosine phosphatase type IVA 1 STRING network

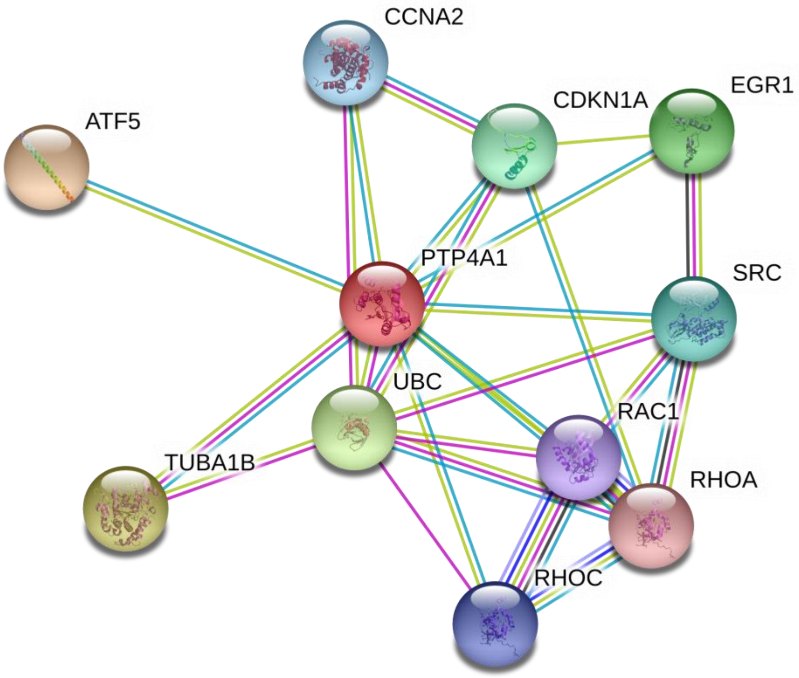

Supplementary Figure S10

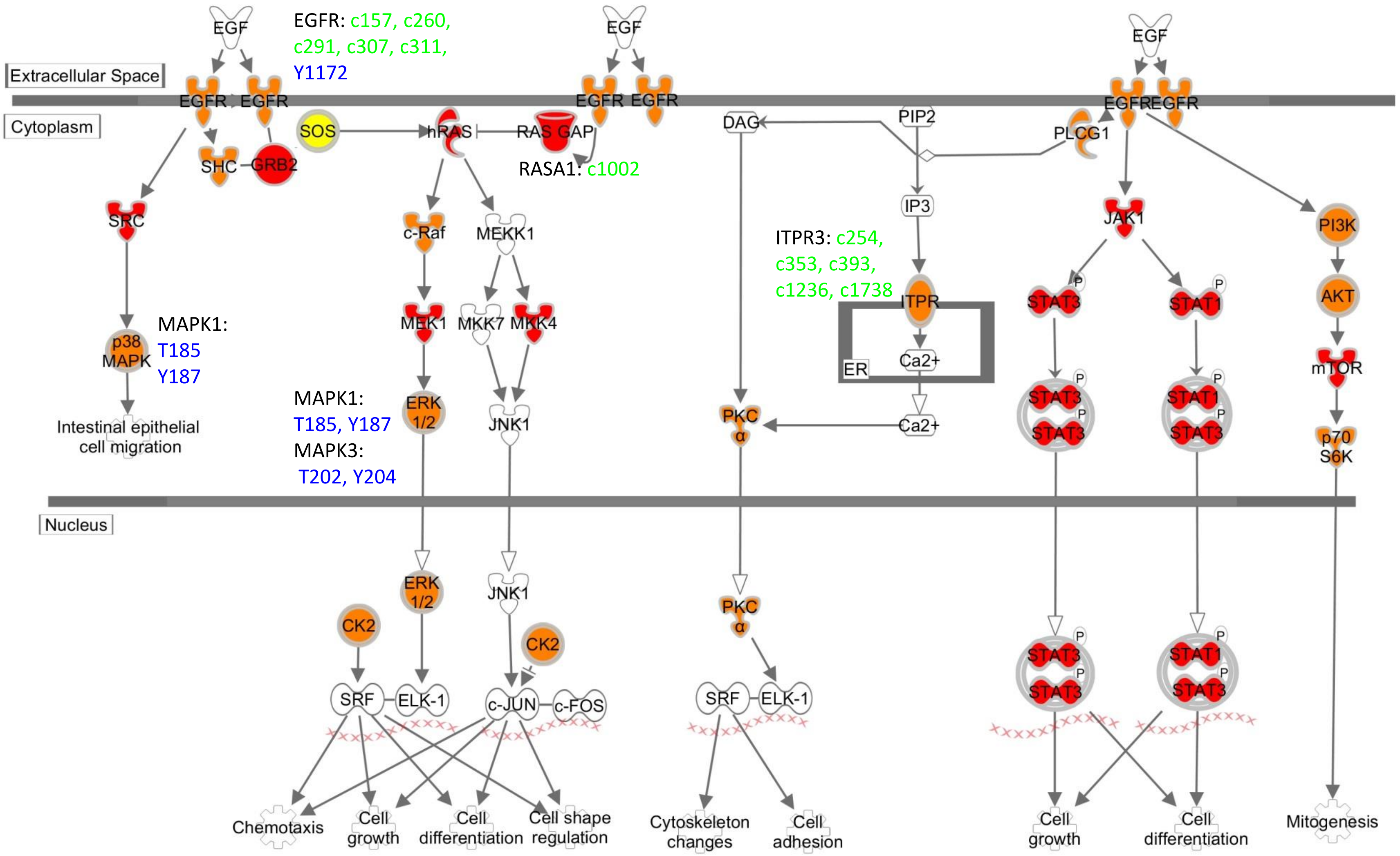

Red: with cys modification
Yellow: with phosphorylation
Orange: with both
Sites in green: downregulated
Sites in blue: upregulated

Supplementary Figure S11

Mitochondrial proteins identified with regulated reversible Cys

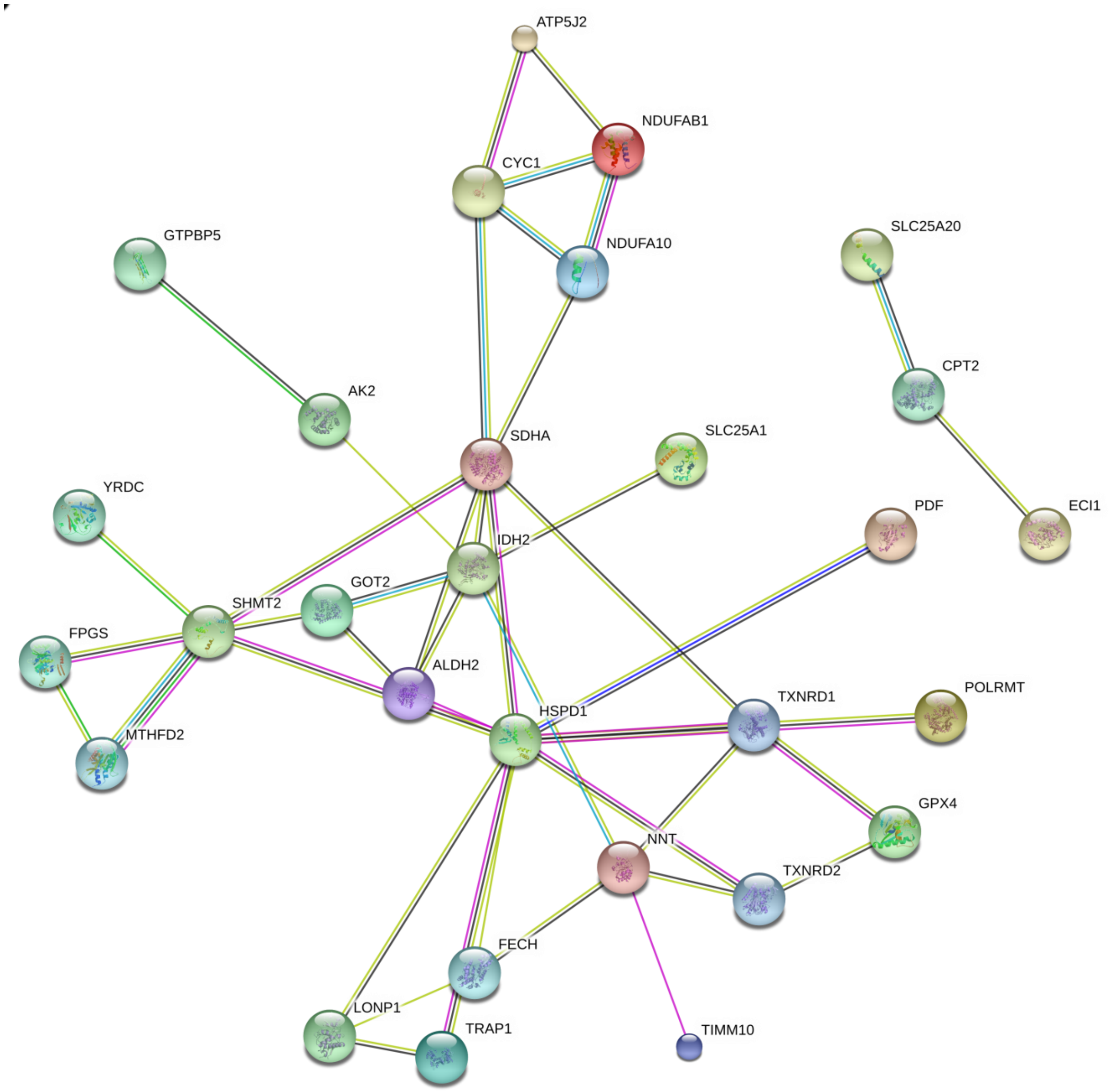

Supplementary Figure S12

Redox related molecules with regulated reversible Cys

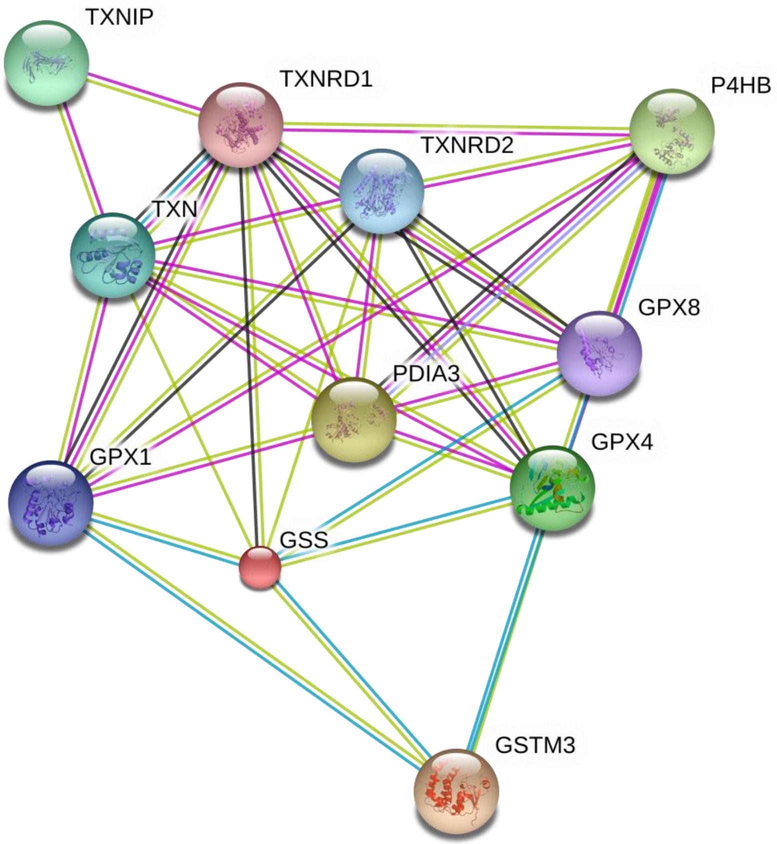

Supplementary Figure S13

Ubiquitinylation and Neddylation related proteins identified with regulated reversible Cys

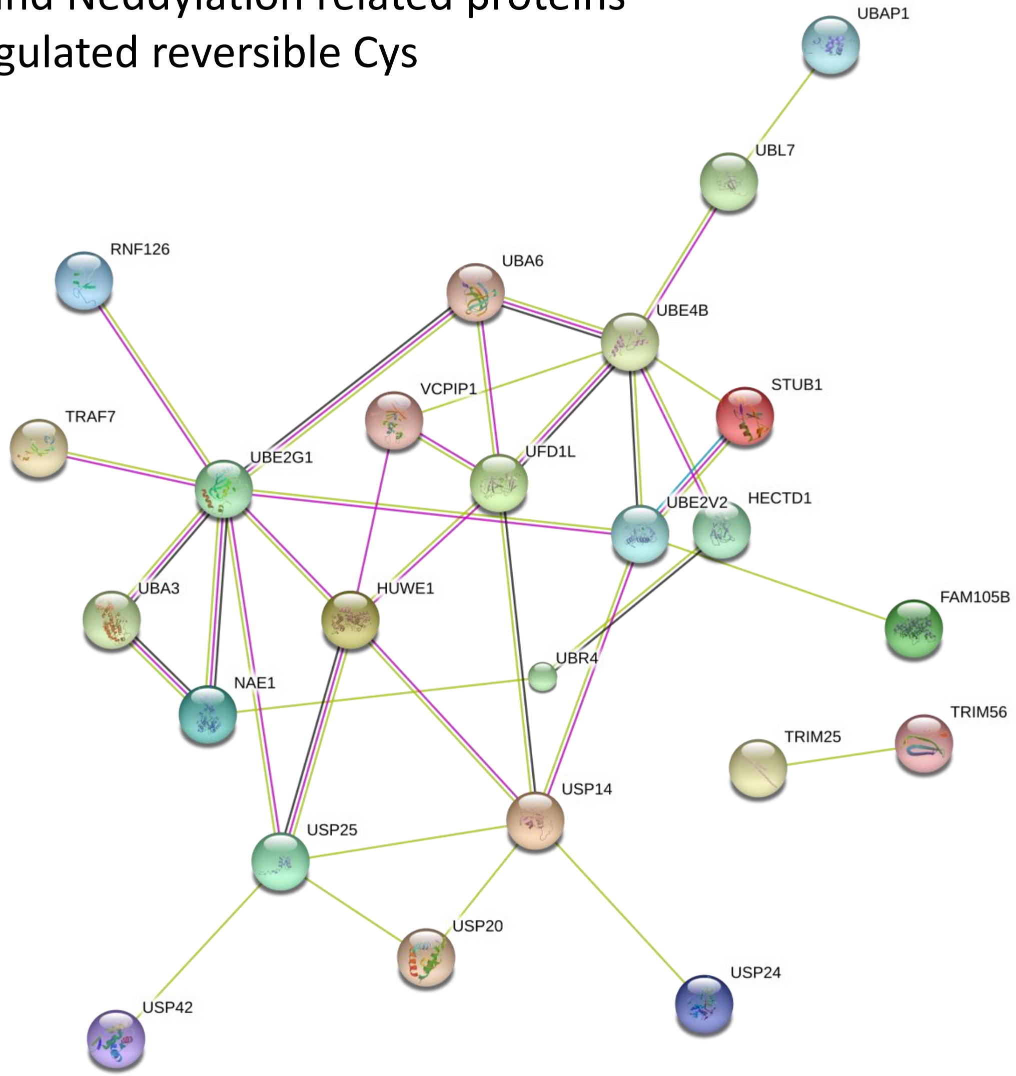

Supplement: Supplemental Data [file 10.1074_M115.054551_mcp.M115.054551-3.pdf]
